# Comprehensive phenotyping and genotyping of a large diverse panel of the wheat wild relative, *Aegilops tauschii* for wheat streak mosaic virus tolerance

**DOI:** 10.3389/fmicb.2025.1723671

**Published:** 2026-01-16

**Authors:** Ved Prakash, Laxman Adhikari, Jesse Poland, Shahideh Nouri

**Affiliations:** 1 Department of Plant Pathology, Kansas State University, Manhattan, KS, United States; 2 Plant Science Program, Biological and Environmental Science and Engineering Division (BESE), King Abdullah University of Science and Technology (KAUST), Thuwal, Saudi Arabia

**Keywords:** *Aegilops tauschii*, GWAS, phenotyping, tolerance, wheat streak mosaic

## Abstract

The wheat streak mosaic (WSM) complex, primarily caused by wheat streak mosaic virus (WSMV) and Triticum mosaic virus (TriMV), results in significant annual yield losses in the northern plains of the United States. Wheat wild relatives, including *Aegilops tauschii*, represent valuable resources of genetic diversity, including resistance to pathogens. In this study, we report the first comprehensive phenotypic assessment and genome-wide association study (GWAS) of a geographically diverse panel of 250 *Ae. tauschii* accessions for WSMV tolerance in single and mixed infections with TriMV. Phenotyping for WSMV symptom severity and quantitative polymerase chain reaction (qPCR)-derived viral titers identified 124 tolerant genotypes in single infections. In double-infection assays, 22 of 39 tested accessions, including both WSMV-tolerant and susceptible genotypes, exhibited tolerance to both viruses. The GWAS revealed that 12 genomic loci were significantly associated with WSMV severity and 8 loci were associated with the viral titer in single infections. Notably, a large effect locus for symptom severity mapped to the long arm of chromosome 5D within lineage two (L2) at 432 Mb. Additional loci in the same region, also identified by the BLINK model, were detected at 430 Mb and 529 Mb. These regions harbor multiple previously reported disease resistance-related genes. These findings suggest that tolerance to WSMV in *Ae. tauschii* is controlled by multiple quantitative trait loci (QTL), highlighting the need for further validation and functional characterization. The WSMV-tolerant germplasm identified in this study constitutes a valuable genetic resource for incorporation into wheat improvement programs. This work lays the foundation for the functional characterization of WSMV tolerance loci in *Ae. tauschii* and provides a framework for leveraging genetic diversity for improving virus resistance in wheat through marker-assisted breeding strategies.

## 1 Introduction

Bread wheat (*Triticum aestivum*), with a hexaploid genome (AABBDD), is one of the major crops grown worldwide with remarkable adaptation to various climatic conditions (Dubcovsky and Dvorak, 2007). With a rapidly growing world population, wheat yield production needs to be increased by 50% over the next few decades to meet the rising food demand (Ray et al., 2013). Pests and pathogens are considered the key yield-limiting factors globally, and among pathogens, viruses are an increasing threat to wheat production and food security.

The wheat streak mosaic (WSM) complex is one of the most economically important viral diseases, which poses a threat to wheat production worldwide (Singh et al., 2018). The disease complex consists of three documented viruses: wheat streak mosaic virus (WSMV), Triticum mosaic virus (TriMV), and High Plains wheat mosaic virus (HPWMoV) (Seifers et al., 2008; Skare et al., 2006; Tatineni et al., 2014; Tatineni and Hein, 2021). WSMV and TriMV are members of the family *Potyviridae,* the genus *Tritimovirus* and *Poacevirus,* respectively. HPWMoV is a type member of the genus *Emaravirus* in the family *Fimoviridae.* All three viruses are transmitted by wheat curl mite, *Aceria tosichella* (Seifers et al., 1997, 2008, 2009; Slykhuis, 1995; Tatineni and Hein, 2018). These viruses can infect wheat in single and mixed infections, producing synergistic effects (Nunna et al., 2025; Tatineni et al., 2010).

To date, only four resistant genes, *Wsm1, Wsm2, Wsm3,* and *c2652,* have been identified, providing different levels of resistance and tolerance to WSMV and TriMV (Danilova et al., 2017; Fahim et al., 2012; Haber et al., 2006; Liu et al., 2011; Lu et al., 2011; Seifers et al., 1995). While *Wsm1* and *Wsm3* show resistance to both WSMV and TriMV (Friebe et al., 1991; Gill et al., 1995; Liu et al., 2011; Wells et al., 1982), *Wsm2* is only resistant against WSMV isolates. *Wsm1* and *Wsm2* genes have been applied in different wheat varieties in the U.S.; however, these resistant genes are temperature-sensitive (Haber et al., 2006; Haley et al., 2002; Seifers et al., 2007). Additionally, a *Wsm2* resistance-breaking strain of WSMV was reported from foxtail (*Setaria viridis*) in the Great Plains in 2019 (Kumssa et al., 2019). Moreover, several recombinant WSMV and TriMV isolates have been identified in the field, increasing the chance of emerging resistance-breaking isolates in wheat (Redila et al., 2021). Therefore, because of the aforesaid challenges, increased effort is warranted to identify and use novel sources of resistance and tolerance against these economically important viruses.

The domestication bottleneck and intense selection have led to a narrow gene pool in bread wheat (Cavanagh et al., 2013). On the other hand, wheat ancestors including *Triticum urartu* (2x; 2n = 14, AA genome), *Aegilops speltoides* (2x; 2n = 14, BB genome), and *Aegilops tauschii* (2n = 2x = 14, DD genome) contain a diverse gene pool and are considered invaluable sources for wheat improvement, including resistance or tolerance to biotic stresses (Feldman and Levy, 2012; Luo et al., 2017; Tanno and Willcox, 2006).

The D genome donor, *Ae. tauschii,* originated from the Caspian Sea region and is distributed across a broad range of Central Asia and has been extensively studied for traits improvement in wheat (Gaurav et al., 2022). *Ae. tauschii* accessions have been divided into two distinct lineages based on their morphology and genetics: lineage 1 (L1), which has been known as subspecies *tauschii*, and lineage 2 (L2), which has been known as subsp. *strangulata* (Singh et al., 2019; Wang et al., 2013). Disease-resistant genes have been identified in this particular species against leaf rust (Cox et al., 1994; Dyck and Kerber, 1970; Kerber, 1987; Lazar et al., 1997; Rowland and Kerber, 1974), stripe rust (Singh et al., 2000), stem rust (Kerber and Dyck, 1978; Marais et al., 1998; Yu et al., 2015), powdery mildew (Lutz et al., 1995; Miranda et al., 2006, 2007), tan spot (Tadesse et al., 2007), septoria tritici blotch (Arraiano et al., 2001), and stem sawfly (Peirce et al., 2024). Despite the significant impact of viral diseases and the great value of genes transferred from *Ae. tauschii* for wheat improvement, this germplasm has not been extensively explored for the existence of sources of resistance or tolerance for the economically important wheat viruses.

Hall et al. (2009) reported a gene conferring resistance to the soil-borne wheat mosaic virus (SBWMV) by screening a segregating recombinant inbred line population with KS96WGRC40 line as one of the parents. This resistance gene within KS96WGRC40, derived from accession TA2397 of *Ae. tauschii,* is located on the long arm of chromosome 5D (Hall et al., 2009). In another study, Nygren et al. (2015) screened a broad range of wheat wild relatives for wheat dwarf virus (WDV) resistance, including a single accession of *Ae. tauschii* (IG46897). Interestingly, virus symptom remission was observed over time in this accession, suggesting that *Ae. tauschii* can be a useful genetic resource for WDV resistance improvement in wheat (Nygren et al., 2015).

In this study, we explored a diverse panel of 250 *Ae. tauschii* accessions for resistance and tolerance to WSMV under single-infection conditions and 39 selected accessions under double-infection conditions with TriMV through combined phenotyping and genotyping. We identified tolerant genotypes, and GWAS analysis revealed that loci were significantly associated with both symptom severity and viral titer.

## 2 Materials and methods

### 2.1 Plant materials

**Figure 1:**
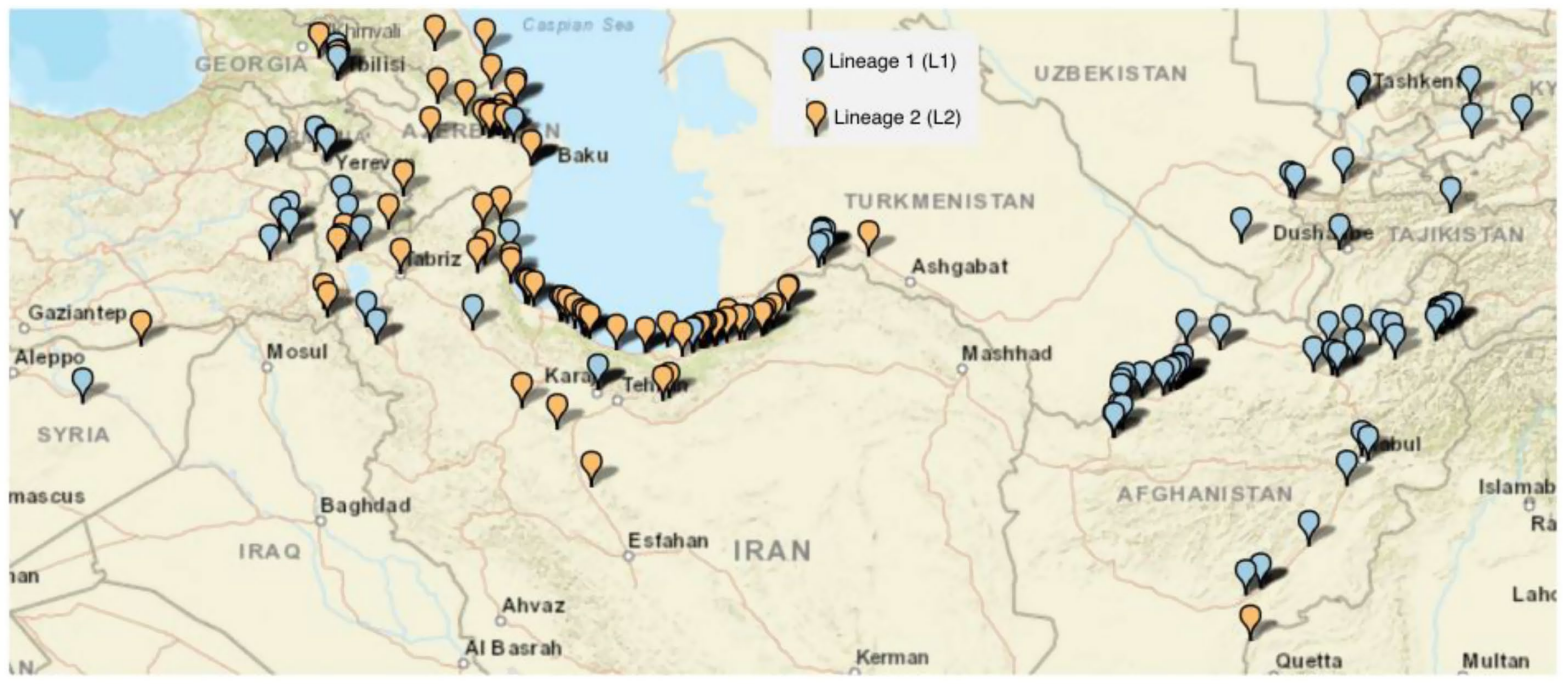
Geographic distributions of *Aegilops tauschii* accessions in the WGS panel. The genetic grouping of the accessions separated the panel into two distinct lineages: lineage 1 (L1) and lineage 2 (L2), each represented by a unique color on the map.

A geographically diverse set of 250 *Ae. tauschii* accessions was explored in this study (Figure 1, Supplementary Table S1). Passport data information of the WSMV screening panel provides detailed information about the accessions’ collection history and origination (Supplementary Table S1). To cover a more diverse pool, accessions from both L1 and L2 were selected for screening. Seeds were obtained from the Wheat Genetics Resource Center (https://www.k-state.edu/wgrc/), and after germination, seedlings were transferred to soil in individual pots in growth chambers with a 16-h light cycle at 23 °C and an 8-h dark cycle at 18 °C.

### 2.2 Virus source and mechanical inoculation

To prepare WSMV inoculum, pUC18: WSMV cDNA infectious clone (Choi et al., 1999) received from Dr. Satyanarayana Tatineni, USDA-ARS, was linearized by enzyme digestion and purified using phenol/chloroform extraction and ethanol precipitation. The purified DNA was used as a template for the synthesis of *in vitro* transcripts using the mMESSAGE mMACHINE™ SP6 Transcription Kit (Invitrogen, CA, USA) as per the manufacturer’s protocol. Due to the unavailability of a cDNA infectious clone, a confirmed infected wheat plant with a TriMV single infection was used as the source of inoculum for TriMV.

To normalize WSMV and TriMV titers, “Tomahawk” wheat seedlings were mechanically inoculated at the two-leaf stage with WSMV transcripts in FES (1% sodium pyrophosphate, 1% bentonite, 1% celite, 0.1 M glycine, and 0.06 M dibasic potassium phosphate) buffer or the sap of TriMV-infected 0.1 M sodium phosphate buffer-grounded leaves. A total of 100 mg of upper non-inoculated (systemic) leaves showing common streak symptoms were collected at 15 days post inoculation (dpi) in 2-ml screw cap tubes for each virus. Leaves were crushed in 1.25 mL of 0.1 M sodium phosphate buffer (pH 7), and 0.25 mL of the lysate was used for RNA isolations, while the remaining 1.0 mL of the lysate was stored at −80 °C.

### 2.3 RNA isolation and RT-qPCR

Total RNAs were isolated using TRIzol LS reagent (Invitrogen, CA, USA) according to the manufacturer’s protocol. The first-strand cDNAs were synthesized using a High-Capacity cDNA Reverse Transcription Kit and random primers (Applied Biosystems, CA, USA) as per the manufacture’s protocol. Standard curves were generated using coat protein (CP) and P3 clones of WSMV and TriMV, respectively, as templates, and SsoAdvanced Universal SYBR Green Supermix (Bio-Rad, CA, USA) with gene-specific primers: WSMV qPCR-F (CCTCGACACGGGAGGAGCTA), WSMV qPCR-R (CGTTGCTCGGCCTCCTGTT), TriMV qPCR-F (GGGAAGCTTCTCAACGAAGG), and TriMV qPCR-R (CCAACTCCCTGAGCGCTGGA). The coordinates for the WSMV primers, referenced to the WSMV genome (Accession No. AF057533.1), are 8,398–8,417 for the forward primer and 8,497–8,479 for the reverse primer. The coordinates for the TriMV primers, referenced to the TriMV genome (Accession No. NC_012799.1), are 3,855–3,874 for the forward primer and 3,963–3,944 for the reverse primer. Viral RNA copy numbers were calculated as previously described (Plumet and Gerlier, 2005).

Samples with similar titers of WSMV and TriMV were selected as the source of inoculum. For double infections, equal amounts of WSMV and TriMV inoculum were mixed and used for inoculation.

### 2.4 Mechanical inoculation

Three-leaf stage *Ae. tauschii* seedlings were mechanically inoculated with either WSMV or WSMV+TriMV inoculum. Buffer-inoculated plants were used as controls. Three to five biological replicates were used for each *Ae. tauschii* genotype tested, in singles as well as co-infections. *Ae. tauschii* TA2341 accession, a previously confirmed WSMV-susceptible genotype, was used as a control for all experiments. Plants were kept in growth chambers for 16:8 h of light:dark cycle at 23:18 °C.

### 2.5 Phenotyping assays

#### 2.5.1 Symptom scoring

Common streak mosaic symptoms were monitored and scored over 1 month (10–31 dpi). Scores were given ranging from 0 to 4, where 0 = no symptom, 1 = light streaking, 2 = mild mosaic and streaking, 3 = severe mosaic and streaking, and 4 = severe mosaic and streaking with yellowing and stunting. To minimize human error, all scorings were conducted by one person. The ggplot2 package was used to generate violin plots and heatmaps for symptoms.

#### 2.5.2 Absolute quantification of viral titers by RT-qPCR

Total RNAs were isolated from 100 mg of upper non-inoculated leaf samples using TRI reagent (Zymo Research, CA, USA) at three time points: 14 (early-infection stage), 21 (mid-infection stage), and 31 (late-infection stage) dpi according to the manufacturer’s protocol. The first-strand cDNAs were synthesized as mentioned above and used as templates for qPCR using SsoAdvanced Universal SYBR Green Supermix (Bio-Rad, CA, USA) and the same gene-specific primers described above. WSMV and TriMV RNA copy numbers were calculated as described above. The ggplot2 package was used to generate violin plots and trends of virus titer in R (version 4.3.1) (Plumet and Gerlier, 2005).

### 2.6 Correlation analysis

To investigate the relationship between symptom and virus titer, Pearson’s correlation coefficient analysis (Pearson and Galton, 1997) was performed for days 14, 21, and 31 dpi using the cor.test() function in R (version 4.3.1). The ggplot2 and ggpubr packages were used to generate scatter plots with fitted linear regression lines and 95% confidence intervals. To assess the strength and statistical significance of the association, annotated values of the correlation coefficient (*r*) and corresponding *p*-values were included in each plot.

### 2.7 Whole-genome sequencing (WGS) and genotyping panel

Whole-genome sequencing (WGS) data for single-nucleotide polymorphism (SNP) marker analysis were primarily obtained from published datasets (Gaurav et al., 2022), comprising all but 17 accessions in our study. The publicly available sequences included accessions designated as BW# identifiers, whose corresponding TA# accessions were cross-referenced using supplementary tables from Gaurav et al. (2022).

For the remaining 17 accessions (TA1598, TA1629, TA1671, TA1704, TA1708, TA2391, TA2415, TA2430, TA2434, TA2448, TA2491, TA2504, TA2535, TA2539, TA2545, TA2548, and TA2552), we performed DNA extraction and whole-genome sequencing (WGS) following the protocols as described in the study Adhikari et al. (2024). Briefly, we prepared PCR-free (350 bp) libraries using the TruSeq DNA PCR-Free LT Sample Prep Kit (Illumina, Inc.), carried out quality control (QC), and conducted paired-end (150 bp) sequencing through Psomagen, Inc. (Rockville, Maryland).

Raw sequencing data were preprocessed using quality trimming with fastp (Chen et al., 2018). High-quality reads were aligned with the *Ae. tauschii* reference genome (assembly GCF_002575655.2, cultivar AL8/78; NCBI BioProject PRJNA341983) using HISAT2 (Kim et al., 2019), and variant calling was performed using bcftools. SNPs were filtered in two steps: (1) variants were filtered based on quality (QUAL ≥30), read depth (DP ≥ 20), allele frequency (0.01 < AF < 0.99), and missing data (≤20%) and (2) we excluded sites with minor allele frequency (MAF < 1%) or heterozygosity (>10%). The resulting high-confidence variant set was used for subsequent genome-wide association study (GWAS) analyses. We split the panel into two genetically distinct subgroups, Lineage 1 and Lineage 2, and ran the GWAS separately for each subgroup while controlling for strong population structure.

### 2.8 Phenotype data analysis for trait values

The WSMV severity scores recorded using repeated plant samples in different batches were analyzed using a mixed model with the ‘*lmer’* function from the lme4 package (R programming language) (R Core Team, 2021). We used genotype as fixed and batch as random. First, we computed means per genotype for repeated observations. Then, we computed the area under disease progress curve (AUDPC) for three different time frames: AUDPC1 from 10 to 15 dpi, AUDPC2 from 15 to 20 dpi, and AUDPC3 from 20 to 31 dpi. We also computed the total AUDPC by summing all three AUDPCs (Jeger and Viljanen-Rollinson, 2001). Then, we computed least squares means (LS-means) for all traits as trait values to run association analysis. Furthermore, the above virus titer quantity measured by qPCR at different dpi (14, 21, and 31) in individual genotypes was used as an additional trait for performing GWAS. The total titer per genotype was obtained by summing the titers across all three dpi.

### 2.9 Population grouping and GWAS

We performed an initial population structure assessment by constructing a phylogenetic tree and conducting principal component analysis (PCA), following the methodology previously described (Adhikari et al., 2022). Genetic distances and other parameters were computed using R packages (dist, ape, phylo, and rrBLUP), and a neighbor-joining (NJ) phylogenetic tree and PCA were generated.

For genome-wide association studies (GWAS), SNPs were encoded as −1, 0, or 1 to fit an additive genetic model. We used FarmCPU and BLINK multi-locus models (Lipka et al., 2012; Wang and Zhang, 2021), implemented in the GAPIT software, to account for population structure complexity. The Q matrix (derived from PCA) was included as covariates. We excluded two accessions from the analysis: a single lineage 3 accession (TA10929) and an admixed accession (TA2482). The GWAS identified loci compared with previous studies and reported the novel and the known loci.

## 3 Results

### 3.1 Screening of *Aegilops tauschii* for WSMV resistance and tolerance in single infections

A total of 250 *Ae. tauschii* accessions were analyzed, representing a broad geographic distribution and including genotypes from both Lineage 1 (L1) and Lineage 2 (L2) (Figure 1).

**Figure 2:**
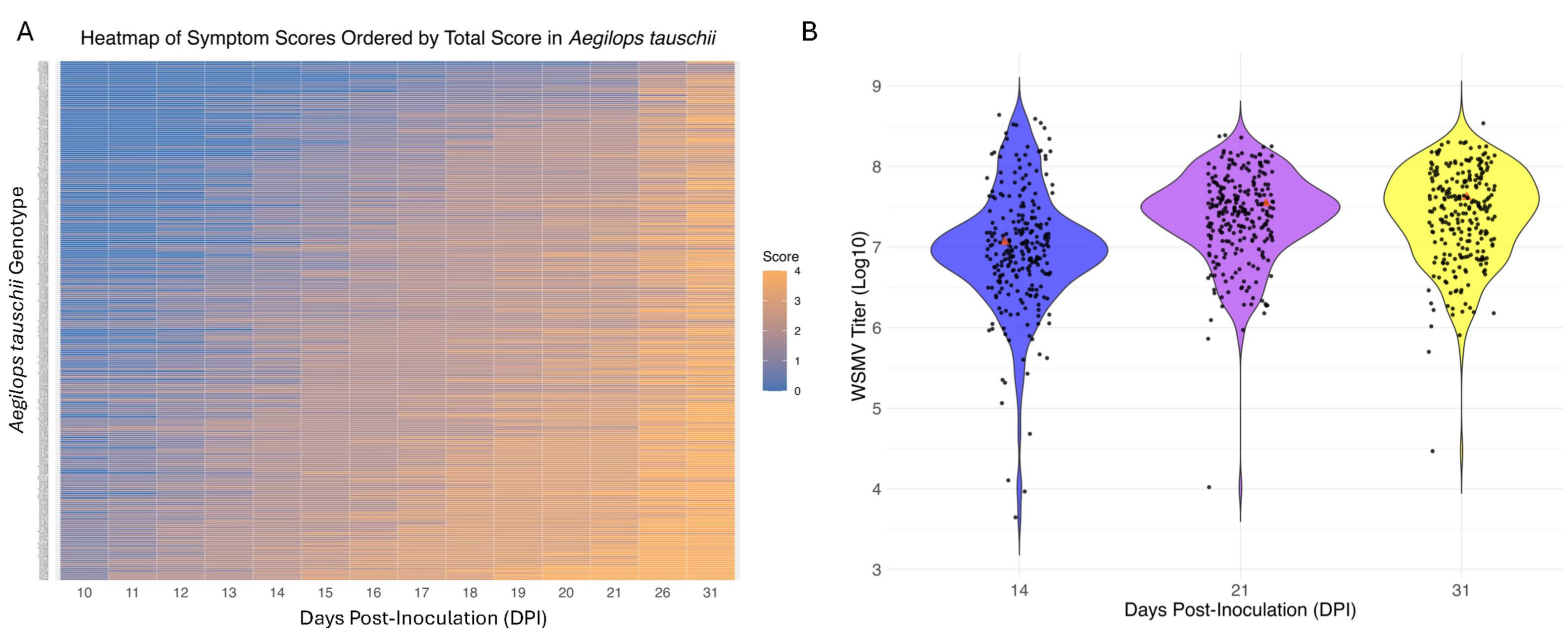
Symptom progression and virus titer distribution in *Aegilops tauschii* genotypes following WSMV infections. **(A)** Heatmap shows temporal progression of WSMV-induced symptoms across 250 *Ae. tauschii* genotypes from 10 to 31 days post inoculation (dpi). Rows represent individual accessions, and columns represent dpi. Symptom severity scores range from 0 (no symptom) to 4 (severe symptom), with a color gradient from blue (low severity) to orange (high severity). **(B)** Violin plots show the distribution of WSMV titers (log_10_-transformed copy number) at 14, 21, and 31 dpi across the same panel of accessions. Each dot represents an individual accession’s titer value at the corresponding time point. The width of each violin indicates the density of data points. Red triangles indicate the reference genotype TA2431 used for comparison in downstream analysis. Both plots were generated using the ggplot2 package in R (version 4.3.1).

The panel was first screened against WSMV in single infections based on symptom severity and qPCR-derived virus titer at three stages of the infection over a period of 31 days (Figures 2, Supplementary Figures 1, 2 and Supplementary Table S1).

#### 3.1.1 Symptom development

All studied *Ae. tauschii* genotypes developed some levels of typical yellow streaks of WSMV symptoms. However, the severity of the developed symptoms varied among genotypes (Figure 2A, Supplementary Figure 1, and Supplementary Table S1), suggesting that different genotypes interact differently with WSMV at different stages of the infection. We targeted 20 dpi to place assessed genotypes in 2 main categories based on symptom development: tolerant (symptom score ≤ 2) and susceptible (symptom score > 2) (Supplementary Table S1). Based on this criterion, 124 *Ae. tauschii* accessions were considered tolerant, while 126 were susceptible.

#### 3.1.2 Virus titer

**Figure 3:**
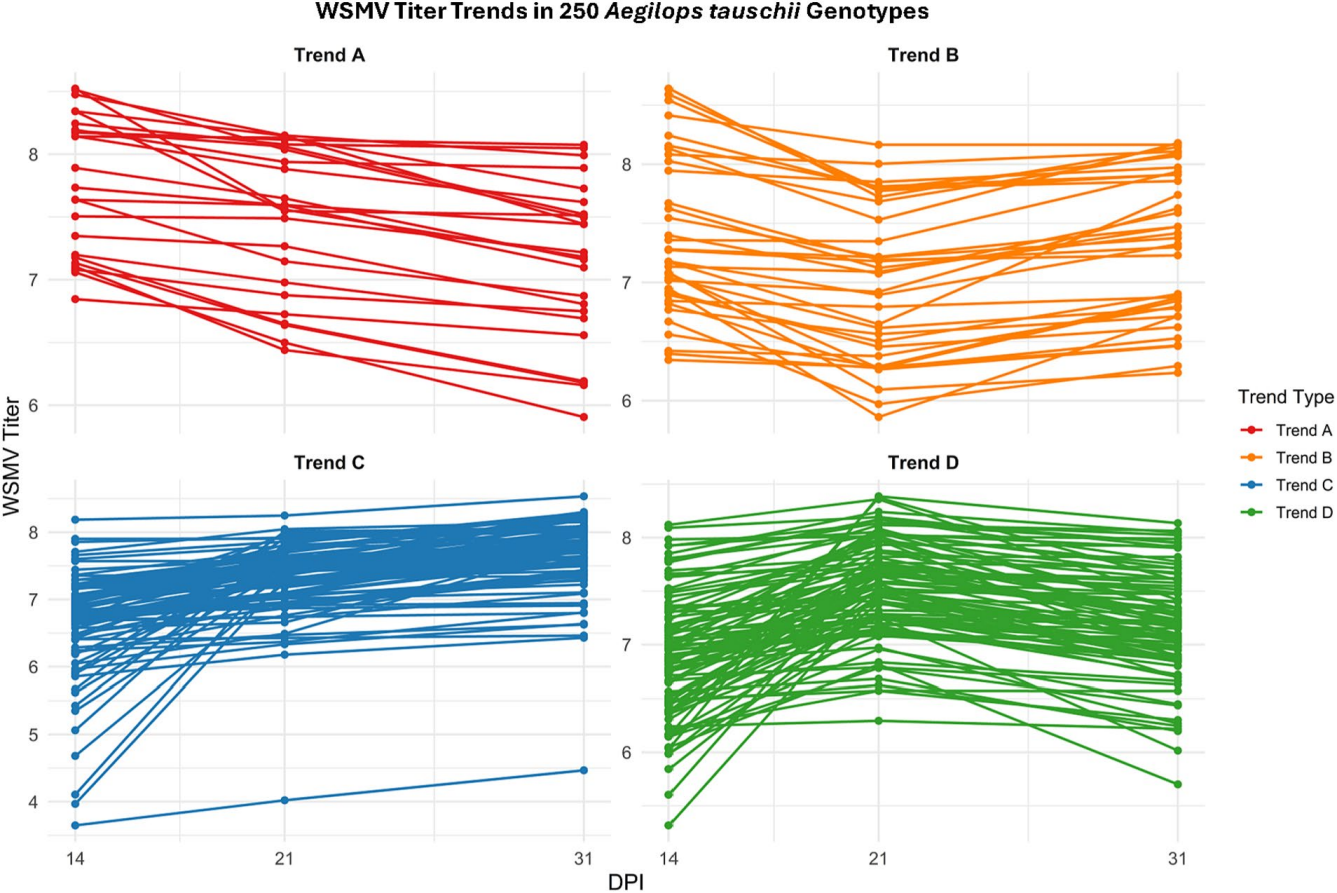
WSMV titer trends in *Aegilops tauschii* genotypes during single infections. Line plots show four major patterns (Trends A–D) of WSMV accumulation across 14, 21, and 31 days post inoculation (dpi) among 250 *Ae. tauschii* genotypes under WSMV infections. Each subplot corresponds to a distinct trend category based on titer trajectories. Each line represents one accession’s WSMV titer, illustrating variation in viral accumulation dynamics. Plots were generated using the ggplot2 package in R (version 4.3.1).

Analysis of the WSMV titer at three infection stages suggested four different trends. In Trend A, we observed a decline in virus replication toward the mid- and late-stages of infection for 29 genotypes (Figure 3). In Trend B, which included 36 genotypes, a reduction in virus titer was observed from early- to mid-infection, followed by an increase from mid- to late-infection stage (Figure 3). A total of 99 genotypes displayed Trend C, in which WSMV accumulation increased gradually from early- to mid- and late-infection stages (Figure 3). Finally, in Trend D (86 genotypes), virus replication was high at the early- and mid-infection with a peak at 21 dpi; however, it decreased to the last stage of the infection (Figure 3).

#### 3.1.3 Symptom development + viral titer

Previous studies placed the tolerance response into “tolerance” and “true tolerance” categories, in which the replication of the pathogen is restricted by the host, resulting in mild symptoms in “tolerance,” while in the “true tolerance” state, the host supports the same level of the pathogen load as in a susceptible plant but has higher yield or quality than the susceptible cultivar (Jeger, 2023; Pagan and Garcia-Arenal, 2020). According to this classification and by considering both symptom scores and viral titers, 119 of 124 WSMV*-*tolerant *Ae. tauschii* fell under the “tolerance” category, while 5 genotypes (TA2463, TA1644, TA2449, TA2393, and TA1579) were considered “true tolerant” (Supplementary Table S1).

**Figure 4:**
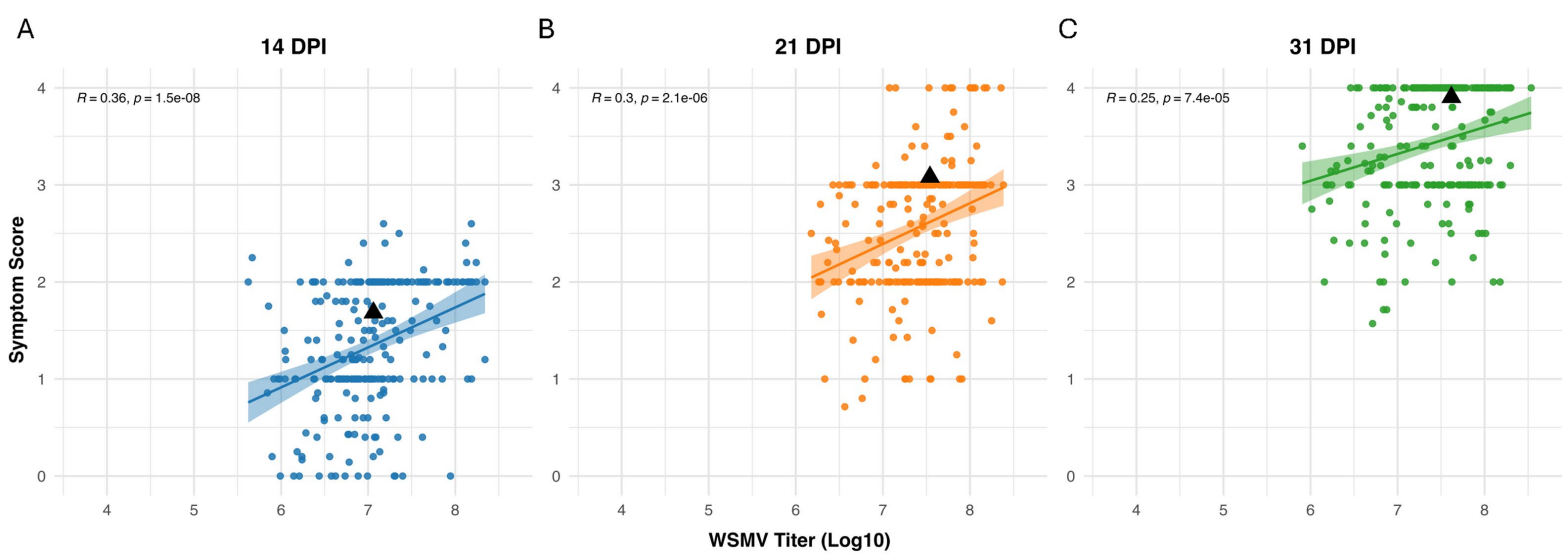
Correlation between WSMV titer and symptom severity in *Aegilops tauschii* genotypes at three infection stages. Positive weak correlation at **(A)** 14 days post inoculation (dpi) (*r* = 0.36, *p* = 1.5 × 10^−8^), **(B)** 21 dpi (*r* = 0.3, *p* = 2.1 × 10^−6^), and **(C)** 31 dpi (*r* = 0.25, *p* = 7.4 × 10^−5^). Scatter plots with fitted regression lines (**A**: 14 dpi, **B**: 21 dpi, **C**: 31 dpi) represent the relationship between log_10_-transformed WSMV titer and symptom scores across accessions. Each point denotes an individual *Ae. tauschii.*

To determine whether there is a correlation between symptom severity and WSMV titer in single infections, we performed Pearson’s correlation analyses at 14, 21, and 31 dpi. Our results revealed a positive correlation between symptom severity and viral titer across all three infection stages, with the strongest correlation at 14 dpi. However, the correlation was relatively weak and declined over time (Figure 4).

### 3.2 Screening of *Aegilops tauschii* in double infections of WSMV and TriMV

We selected 29 *Ae. tauschii* genotypes from the above WSMV-tolerant panel, along with 10 susceptible genotypes, for a total of 39 for screening in double infections of WSMV and TriMV. The selection of these 29 WSMV-tolerant genotypes was based on their consistently low symptom score (<3) through the majority of the infection period.

#### 3.2.1 Symptom development

**Figure 5:**
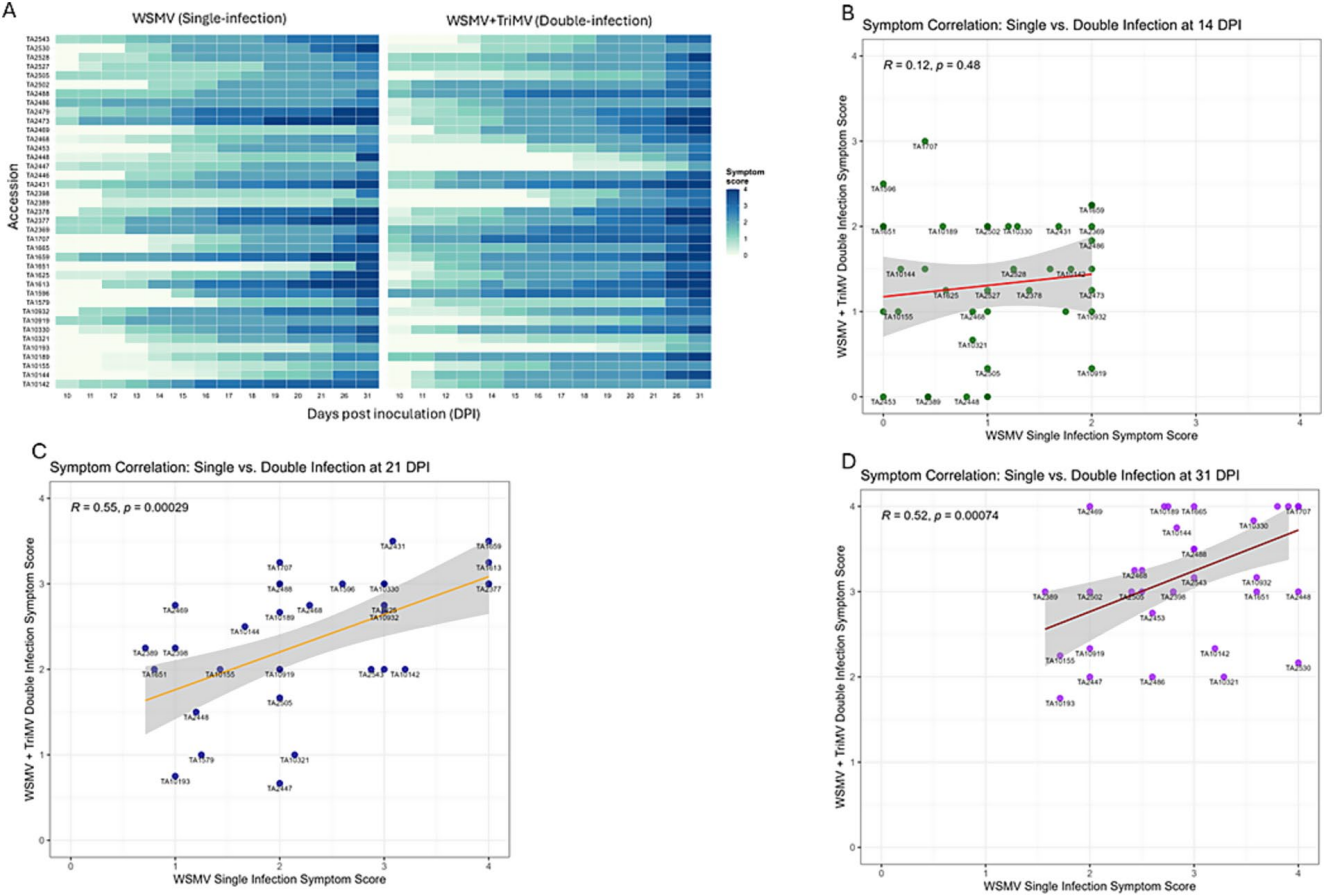
Symptom progression in *Aegilops tauschii* genotypes under single- and double-infection conditions. **(A)** Heatmap compares the symptom scores in common genotypes during WSMV single and double infections with TriMV. **(B–D)** Mean symptom score comparison under WSMV single infection versus double infection with TriMV at 14, 21 and 31 days post inoculation (dpi), respectively. Values shown are model-adjusted means calculated from a linear mixed-effects model. Shaded areas show ± standard error around the mean curves. Plots were generated using the ggplot2 package in R (version 4.3.1).

All 39 genotypes developed typical yellow streak mosaic symptoms of wheat streak mosaic (WSM) complex (Figure 5A, Supplementary Figure S3, and Supplementary Table S2). Using the same criteria as for single infections, 22 of the 39 genotypes were classified as tolerant and 17 as susceptible under double-infection conditions (Supplementary Table S3). Surprisingly, the genotype TA10142, which was susceptible to WSMV single infection, exhibited a tolerance response under co-infection. This was unexpected because synergistic interactions between WSMV and TriMV have been reported in wheat.

Conversely, genotypes TA1596, TA1707, TA2369, and TA2488, which were tolerant to WSMV single infections, lost this tolerance under co-infections with TriMV, indicating that the synergistic interaction between WSMV and TriMV overcame the tolerance.

Comparison of symptoms between single and double infections showed that they largely overlapped around mid-infection (Figures 5B,C). However, symptom severity increased over time in double infections in 38% of genotypes (Figure 5C), consistent with the synergism between WSMV and TriMV previously reported by Tatineni et al. (2019) in wheat. In 28% of genotypes, symptom severity was higher in WSMV single infections, while the remaining 34% showed mixed responses (Figures 5B,C).

#### 3.2.2 Virus Titer

**Figure 6:**
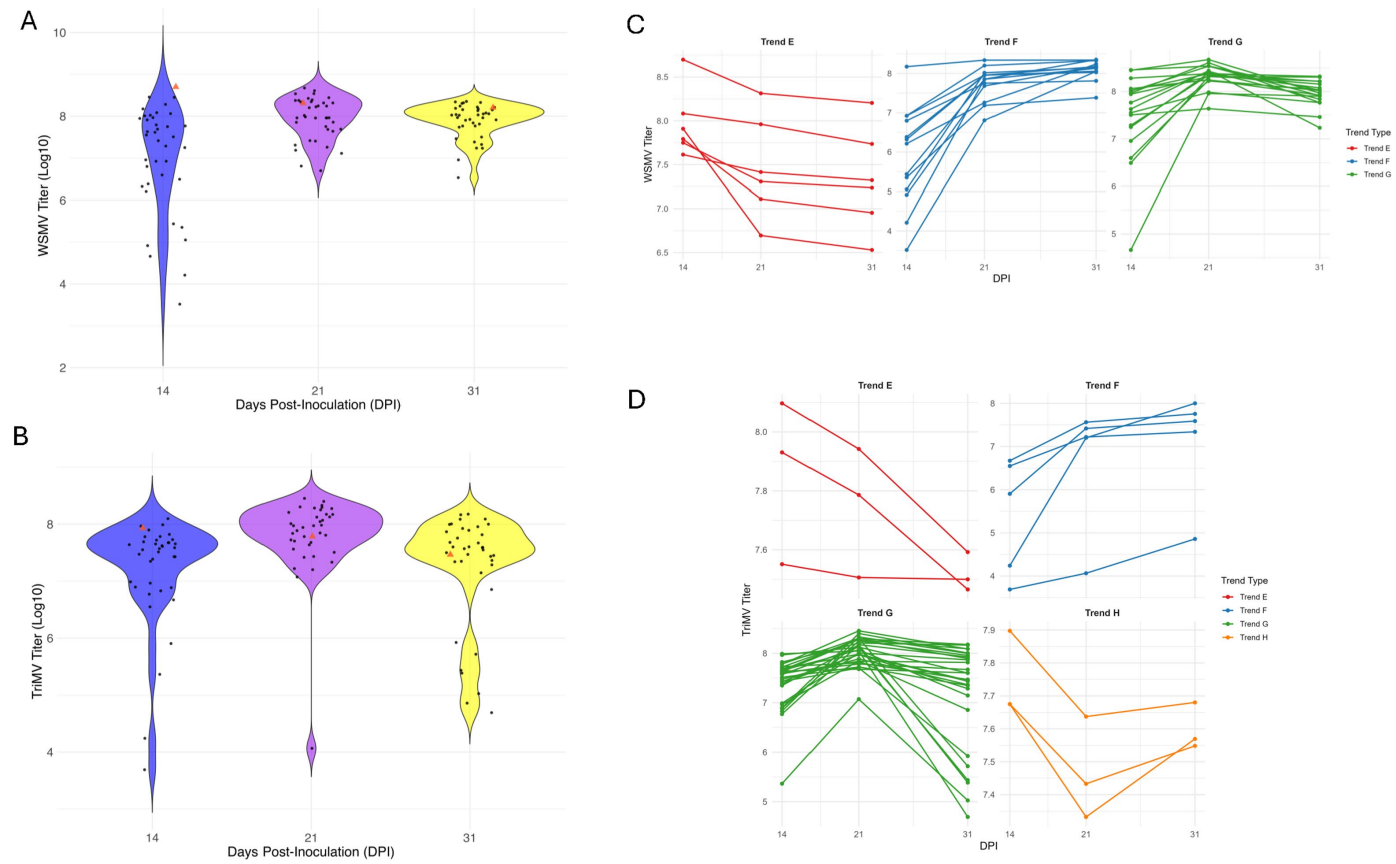
Viral titer distributions and titer progression trends across *Aegilops tauschii* genotypes at 14, 21, and 31 days post inoculation (dpi) under double-infection conditions. Viral load from early- to late-stages of infection for WSMV **(A)** and TriMV **(B)**. Each dot represents virus titer measured in an individual genotype. The width of each violin reflects the kernel density of the data. Blue denotes viral titer at 14 dpi, purple denotes viral titer at 21 dpi, and yellow denotes viral titer at 31 dpi. Line plots represent individual accessions grouped by distinct viral accumulation patterns (Trends) over three time points (14, 21, and 31 dpi) for WSMV **(C)** and TriMV **(D)**. Plots were generated using the ggplot2 package in R (version 4.3.1).

We measured the titer of both WSMV and TriMV at 14, 21, and 31 dpi in 39 screened accessions (Figures 6A,B and Supplementary Table S2). Similar to WSMV single infection, in this study, we also observed several trends (Trends E-H) for viral titer changes over time. Trend E, characterized by a decrease of the titers of both viruses over time, included six and three genotypes for WSMV and TriMV, respectively (Figures 6C,D). Trend F (18 genotypes: 13 for WSMV and 5 for TriMV) displayed steadily increasing viral titers through the infections (Figures 6C,D). Trend G (18 genotypes for WSMV and 28 genotypes for TriMV) showed titers that rose to a peak at 21 dpi before declining (Figures 6C,D). Trend H, the opposite of Trend G, exhibited an initial (early- to mid-infections) decrease in viral titer followed by a late-stage increase. This later trend was observed in one and three genotypes for WSMV and TriMV, respectively, making it, along with Trend E, one of the less common patterns (Figures 6C,D). Trend G was the most prevalent among the observed trends, especially for TriMV.

**Figure 7:**
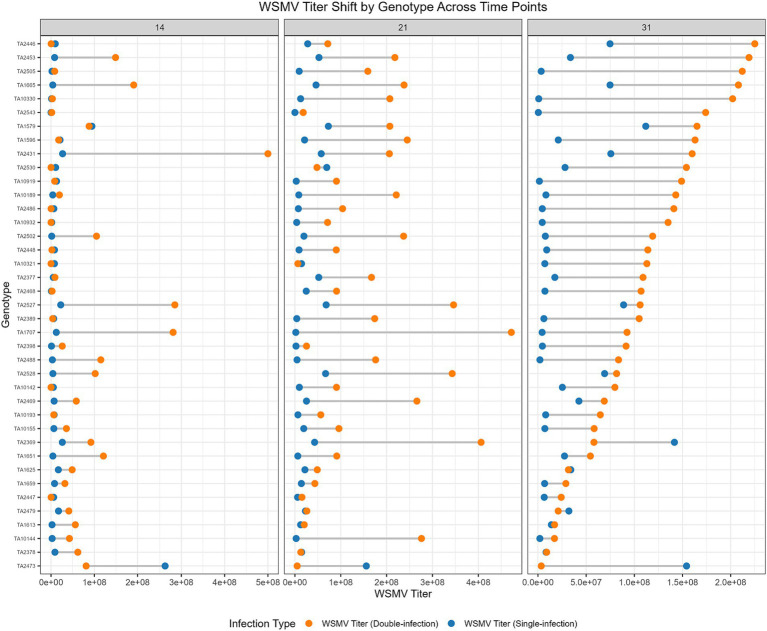
WSMV titer comparison in single infection vs. double infection at 14, 21, and 31 dpi. Genotypes are sorted based on the 31 dpi titer values.

WSMV titers were relatively higher in mixed infections than in single infections, suggesting enhanced replication of WSMV in the presence of TriMV (Figure 7 and Supplementary Figures 4A,B).

#### 3.2.3 Symptom development and viral titer

Considering both symptom development and viral titers, genotype TA10193 displayed the lowest TriMV accumulation across all three time points, moderate WSMV accumulation, and a symptom score < 2, suggesting potential natural tolerance to co-infections. Similarly, TA10321 showed the lowest WSMV accumulation at 14 and 21 dpi but consistently high TriMV titers while maintaining a symptom score of 2 until the late-stage of infection, indicating another genotype with a good level of natural tolerance to co-infections.

Genotypes TA10155 and TA1651 showed consistently lower WSMV titers but higher TriMV titers from 14 to 31 dpi while maintaining lower symptom severity than the positive control, TA2431. This suggests that both genotypes are tolerant to co-infections, in addition to their tolerance to WSMV single infections (Figures 5A,C and Supplementary Tables S2, S3).

Symptoms were delayed in genotypes TA1579, TA10193, TA10321, TA2389, TA2398, TA2453, and TA2448 under both single- and double-infection conditions, suggesting that these genotypes, along with TA10155 and TA1651, represent the most tolerant genotypes to double infections (Figures 5A,C and Supplementary Tables S2, S3).

**Figure 8:**
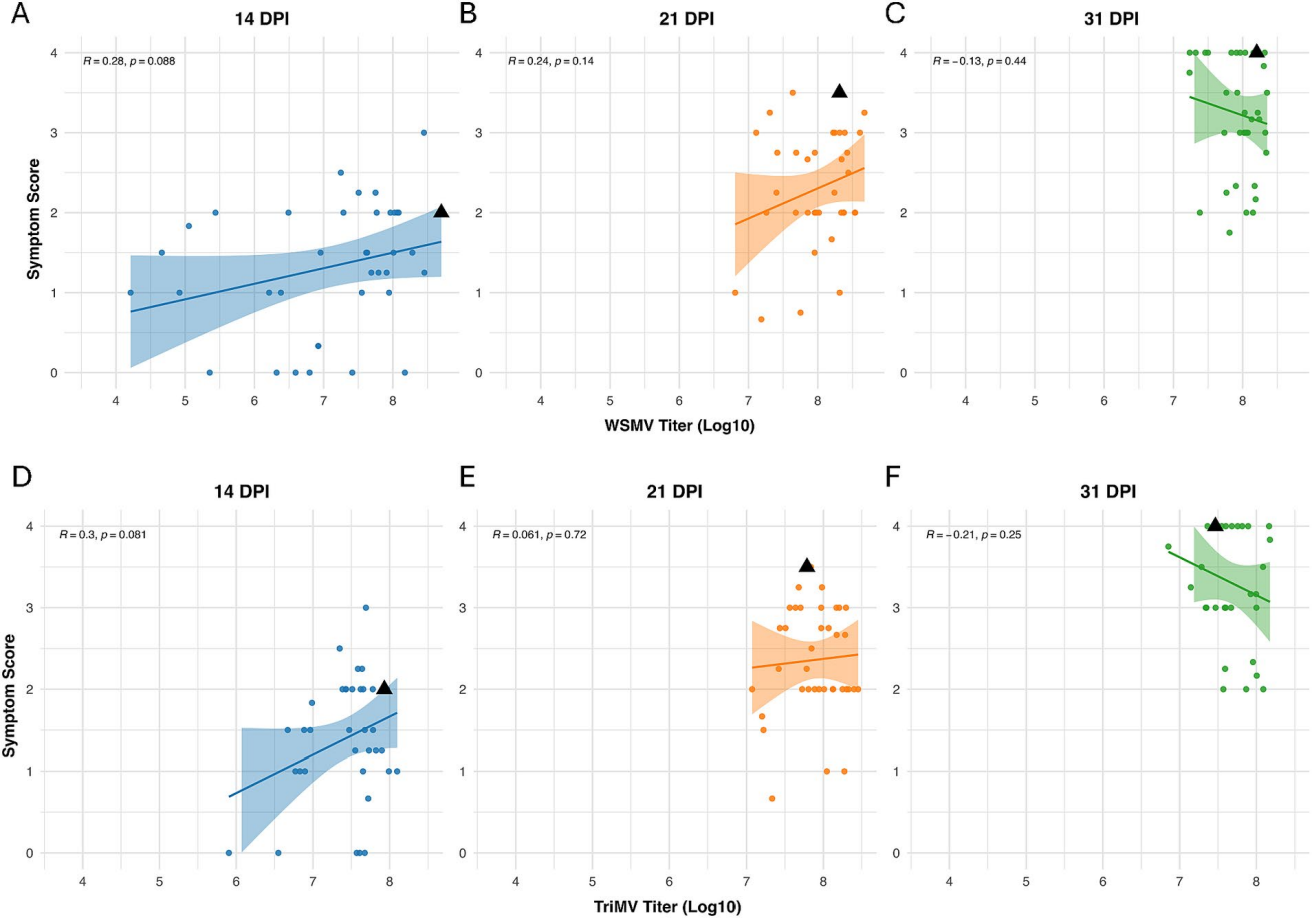
Correlation analysis between WSMV and TriMV titer (log₁₀-transformed) and symptom scores across three infection stages in *Aegilops tauschii* under double-infection conditions. WSMV titer vs. symptom score at **(A)** 14, **(B)** 21, and **(C)** 31 dpi. TriMV titer vs. symptom score at **(D)** 14, **(E)** 21, and **(F)** 31 dpi. Each scatterplot shows a fitted regression line with a 95% confidence interval. A weak positive correlation is observed between viral titers and symptom severity for both viruses at 14 and 21 dpi.

Pearson’s correlation analyses indicated a weak but positive correlation between symptom enhancement and WSMV or TriMV titers at early- and mid-infection stages (Figure 8), with correlation declining at later stages of the infection, possibly due to symptom saturation or overlapping viral dynamics. The strongest correlation was observed for TriMV at 14 dpi (*r* = 0.3), suggesting that TriMV may be the primary contributor to early symptom expression during co-infections with WSMV.

### 3.3 SNP genotyping and population subgrouping

Given the limited number of *Ae. tauschii* accessions screened under double infections, our genomic analyses focused only on WSMV single infections. We identified and retained 93,089,447 SNPs after two rounds of filtration (Supplementary Table S4). For the initial population analyses—including population structure and PCA—we used a random 2% subset (1,870,696 SNPs) of the filtered SNPs (Supplementary Table S4).

We observed strong population structure, as revealed by the neighbor-joining (NJ) tree and principal component analysis (PCA) plot (Supplementary Figure S5), consistent with previous studies. To minimize false positives caused by population structure effects on linkage disequilibrium (LD), we conducted GWAS separately for the two genetic lineages, Lineage 1 (L1) and Lineage 2 (L2). For L1 and L2, we obtained 447,438 and 728,899 biallelic SNPs, respectively, from the filtered genotyping file containing all samples (Supplementary Table S4).

### 3.4 Phenotypic variation

Significant variation in AUDPC values (AUDPC1, AUDPC2, AUDPC3, and total AUDPC) was observed across the screening panel (Supplementary Figure S6). Specifically, AUDPC1—calculated for early-infection (10–15 dpi)—ranged from 0 to 10.55; AUDPC2—calculated for mid-infection (15–21 dpi)—ranged from 0.6 to 17.2; AUDPC3—calculated for late-infection (21–31 dpi)—ranged from 12.7 to 43.8; and total AUDPC ranged from 13.7 to 70.5 (Supplementary Table S5; Supplementary Figure S6). Accessions TA1579, TA1651, and TA2548 exhibited zero values for AUDPC1, indicating early-infection stage tolerance to WSMV (Supplementary Table S5). Notably, TA1651 displayed the lowest total AUDPC value (13.7), positioning it as a promising candidate for resistance/tolerance breeding. Both TA1579 and TA1651 exhibited sustained tolerance under double-infection conditions (Figure 5C and Supplementary Table S3). The majority of AUDPC values in Lineage 1 and Lineage 2 followed normal to near-normal distributions (Supplementary Figure S6). Additionally, considerable genetic variation in virus titer levels was observed among the tested genotypes (Supplementary Figure S7).

We found a positive moderate to low correlation between AUDPC and WSMV titer. The highest correlation (0.36) was obtained between the total AUDPC and titer at 14 dpi (Supplementary Figure S8).

### 3.5 Genome-wide association study

#### 3.5.1 Loci associated with WSMV severity

**Figure 9:**
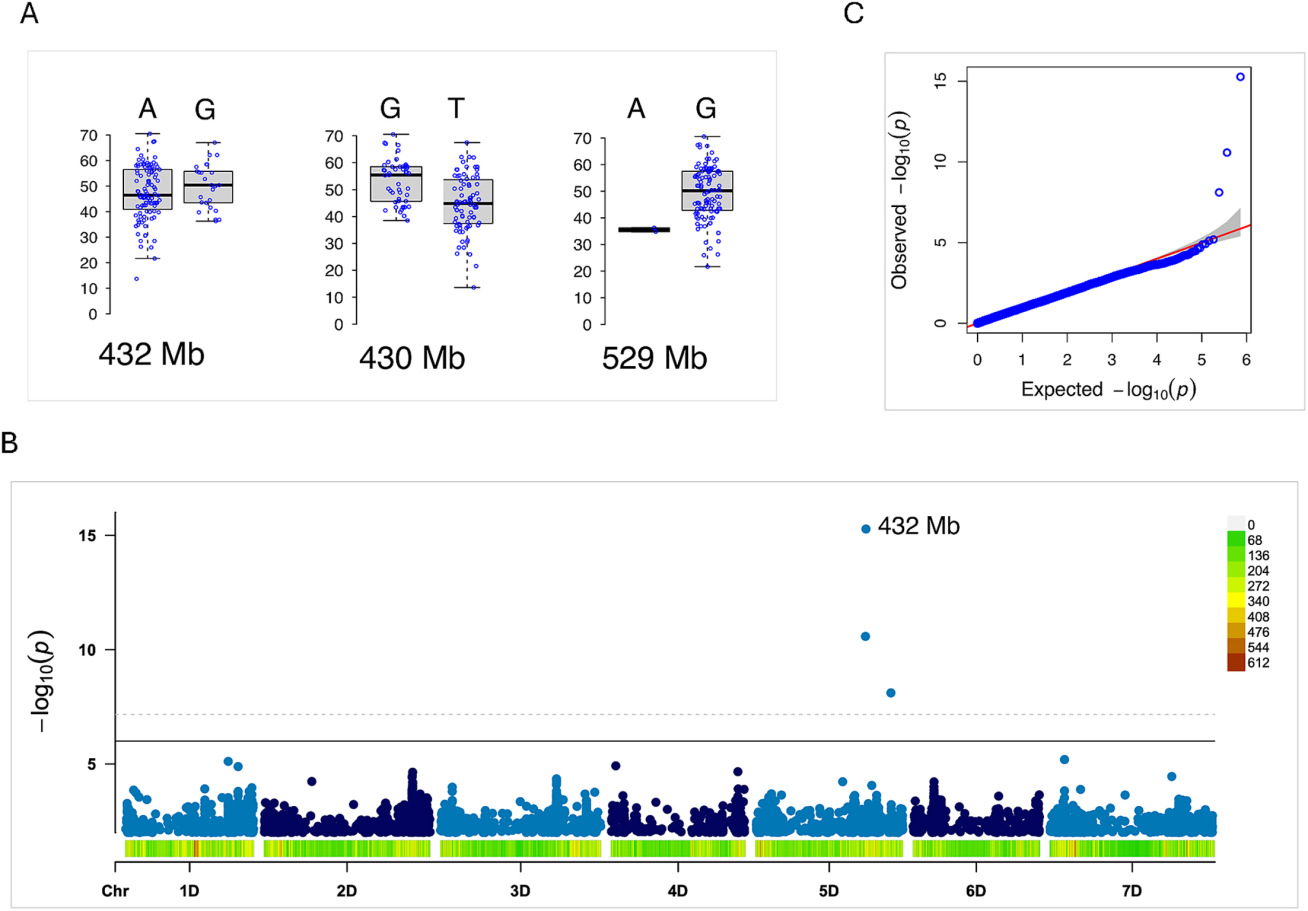
Genome-wide association study (GWAS) for WSMV symptom severity in the *Aegilops tauschii* mapping population (Lineage 2). **(A)** Phenotypic distribution of WSMV symptom severity score for the two alleles at the most significant loci at 430, 432, and 529 Mb on chromosome 5DL. **(B)** Manhattan plot highlights the significant locus on chromosome 5D. **(C)** Quantile–quantile (QQ) plot of *p*-values obtained using the BLINK method.

In this study, we identified 12 significantly associated loci for WSMV severity based on AUDPC values in *Ae. tauschii* with different levels of statistical significance (*p* values) (Table 1). The most significant loci were located on the long arm of chromosome 5DL (Table 1 and Figure 9). Both the BLINK and FarmCPU models detected associated SNPs for total AUDPC at 430 Mb and 432 Mb on chromosome 5DL within the Lineage 2 population. Additionally, the BLINK model identified another WSMV severity-associated locus at 529 Mb on 5DL, highlighting this chromosomal region as a promising target for further study (Figure 9).

**Table 1:** Genomic regions that are statistically significant (*p* < 0.05) after Bonferroni correction or suggestive threshold (1e-6) for WSMV severity scores and virus titer quantity measured at different dpi.

Traits	SNP at peak	GWAS model	*P*-value	Population
audpc	5D_432104216	Blink and FarmCPU	5.30e-16	Lin2
audpc	5D_430169946	Blink and FarmCPU	2.6e-11	Lin2
audpc	5D_529643816	Blink	7.7e-09	Lin2
audpc	1D_493175253	FarmCPU	2.5e-09	Lin2
audpc	1D_26167097	Blink	3.47e-08	Lin2
audpc	3D_145555846	Blink and FarmCPU	2.3e-07	Lin1
audpc1	6D_493922456	Blink and FarmCPU	5.7e-11	Lin2
audpc1	2D_31532197	Blink	7.2e-06	Lin2
audpc2	6D_493922456	FarmCPU	1.8e-13	Lin2
audpc2	5D_368961	Blink	5.5e-14	Lin2
audpc2	1D_26167097	Blink	3.47e-08	Lin2
audpc3	2D_602887587	Blink	1.5e-10	Lin2
Titer_21.dpi	2D_25565245	Blink and FarmCPU	3.3e-25	Lin1
Titer_21.dpi	1D_50876748	Blink	3.5e-17	Lin1
Titer_21.dpi	1D_382955750	FarmCPU	7.2e-12	Lin1
Titer_21.dpi	5D_131768902	FarmCPU	1.6e-16	Lin1
Titer_21.dpi	5D_357321658	Blink and FarmCPU	1.3e-13	Lin2
Titer_14.dpi	4D_108049137	Blink	3.1e-11	Lin2
Titer_21.dpi	7D_125252267	Blink and FarmCPU	1.1e-10	Lin2
Titer_21.dpi	7D_42944374	FarmCPU	5.3e-09	Lin1

The *p*-values are reported for the GWAS models. The loci naming follows the format chromosome_position. For the same loci identified by the two models, we used the *p*-value of the model that is in bold letters.

We also detected two loci for total AUDPC on chromosome 1DL (at 26 Mb and 493 Mb) and one locus on chromosome 3DS (at 145 Mb). Notably, the locus on 3DS (145 Mb) was the only region identified in the Lineage 1 population, indicating differential genetic responses to WSMV infections between the two *Ae*. *tauschii* lineages (Table 1). A SNP on chromosome 6DL (at 493 Mb) and another on 2DS (at 31.5 Mb) were associated with AUDPC1. For AUDPC2, we identified three loci, whereas a single locus on 2DS was linked to AUDPC3 (Table 1). Two loci overlapped across different datasets: the locus at 1DS (at 26 Mb) was identified for both total AUDPC and AUDPC2, and the locus on 6DL overlapped for AUDPC1 and AUDPC2 (Table 1).

#### 3.5.2 Potential loci associated with virus titer

**Figure 10:**
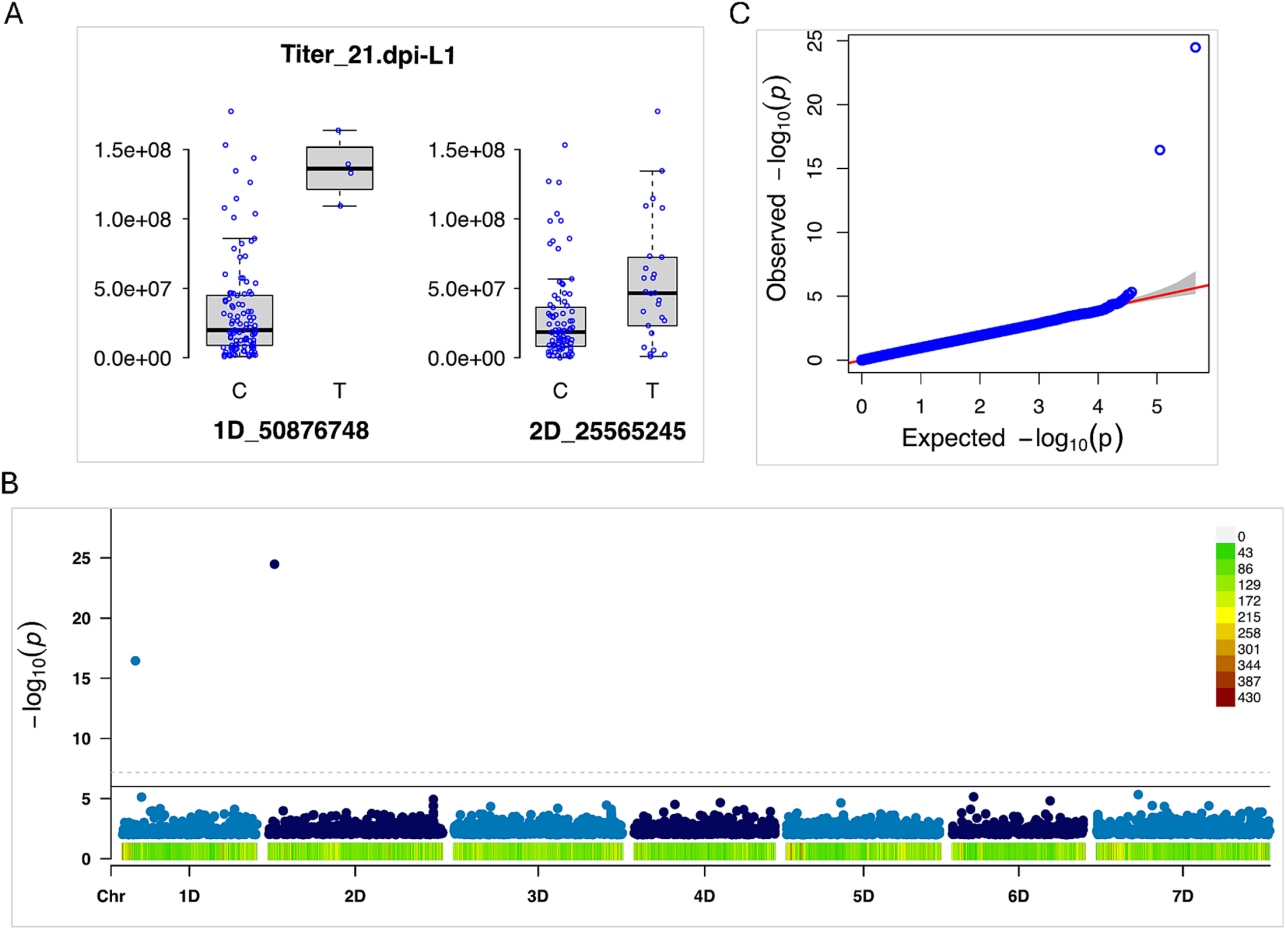
Genome-wide association study (GWAS) for WSMV titer accumulation in the *Aegilops tauschii* mapping population (Lineage 1). **(A)** Phenotypic distribution of WSMV titer at 21 days post inoculation (dpi), shown for the two alleles at the significant loci on chromosomes 1D and 2D. **(B)** Manhattan plot highlights significant loci on chromosomes 1D and 2D. SNP density across the chromosomes is indicated by the color scale along the *x*-axis; the inset (top right) shows the corresponding color legend. **(C)** Quantile–quantile (QQ) plot of *p*-values from the BLINK method.

Eight loci were significantly associated with virus titer quantity (Table 1 and Figure 10). These loci were located on chromosomes 1DS, 2DS, 4DS, 5DS, 5DL, and 7DS. Five loci were detected in the L1 subpopulation, and three loci were detected in the L2 subpopulation. Of the eight loci, seven loci were detected for Titer_21.dpi, and the remaining one was identified for Titer_14.dpi. Interestingly, the loci associated with the titer amount were different than the loci associated with the symptom severity score based on AUDPC (Table 1).

#### 3.5.3 Existing and novel loci and validation

In this study, we identified a highly significant locus on chromosome 5DL at ~430 Mb (Table 1). This region harbors multiple disease resistance-related genes, including *RGA4* and *RGA5* (~429 Mb), two nucleotide-binding and leucine-rich repeat domain proteins (NB-LRRs), and several protein kinases (NCBI Assembly GCF_002575655.2). We surveyed the 5DL QTL region from 425 Mb to 435 Mb and found 194 characterized and uncharacterized genes (Supplementary Table S6). These findings highlight previously uncharacterized loci that warrant fine mapping and functional validation.

## 4 Discussion

The D genome in the modern bread wheat originated primarily from Ae. tauschii populations found in the southwestern Caspian Sea region (Cavalet-Giorsa et al., 2024). Many resistance genes against biotic and abiotic stresses have been reported in *Ae. tauschii* accessions (Carrera et al., 2012; Jin et al., 2024; Miranda et al., 2006; Pourkhorshid et al., 2022; Suneja et al., 2019; Yoshioka et al., 2024; Yu et al., 2015). However, this valuable genetic resource has remained largely unexplored for economically important wheat viral diseases. In this study, we performed a comprehensive phenotypic screening and GWAS of nearly the full panel of *Ae. tauschii* accessions, revealing substantial natural variation in tolerance to WSMV and TriMV. These findings highlight the potential of *Ae. tauschii* as a source of novel tolerance alleles and provide critical insight for future wheat improvement programs targeting viral disease management.

While we did not find any complete resistance response to WSMV among studied *Ae. tauschii* accessions, we reported 124 WSMV-tolerant genotypes in single infections. While the majority of germplasm screenings for plant viruses rely on symptom development and/or the presence or absence of the virus as determined by enzyme-linked immunosorbent assay (ELISA) (Hall et al., 2009; Makkouk et al., 1994; Nygren et al., 2015), we quantified absolute viral replication titers in each infected plant at three stages of the infection: early, mid, and late by real-time PCR. Phenotyping through viral titer measurement helps to select low or virus-free genetic lines, especially in breeding programs. By combining symptom severity data and viral replication titers, we were able to identify genotypes (TA2463, TA1644, TA2449, TA2393, and TA1579) that were “true tolerant” because, despite high levels of WSMV titer, infected plants displayed low symptom severity during virus infection (Supplementary Table S1). Hence, we believed that this approach provides us with more precise and detailed information about the virus infection status and the host response at various stages of the infection. This large-scale screening of a wheat wild relative, based on both the symptom severity and virus titer at three main stages of the virus infection, has the potential to uncover many novel virus resistance genes and novel mechanisms of tolerance.

Our screening included genotypes TA1618, TA1695, and TA1582, previously reported as resistant to wheat curl mite (WCM) (Gaurav et al., 2022; Malik et al., 2003), the natural vector of WSMV and TriMV. However, all three genotypes were susceptible to WSMV under single-infection conditions (Supplementary Table S1). Additionally, our screening identified TA2460 as a WSMV-tolerant genotype, which has also been reported to carry the leaf rust resistant genes *Lr41* (Cavalet-Giorsa et al., 2024; Lazar et al., 1997; Rudd et al., 2014).

Under WSMV single–infection conditions, symptom severity was positively correlated with viral load at all time points, with the strongest correlation observed at 14 dpi (14 dpi: *r = 0.36, p = 1.5 × 10^−8^*; 21 dpi: *r = 0.3, p = 2.1 × 10^−6^*; 31 dpi: *r = 0.25, p = 7.4 × 10^−5^*). The strength of the correlation declined over time (Figure 4). To assess whether the *Ae. tauschii* WSMV-tolerant genotypes identified in this study could maintain tolerance under double-infection conditions with TriMV, we selected a subset of genotypes from the WSMV-tolerant panel and screened them for both viruses. We identified 22 accessions that retained tolerance to both WSMV and TriMV under double-infection conditions (Figure 5A and Supplementary Table S3); however, no genotype showed a “true tolerance” response under double infections. Surprisingly, TA10142, which was susceptible to WSMV alone, exhibited tolerance under double-infection conditions. This unexpected outcome, despite reported synergism between WSMV and TriMV in wheat (Nunna et al., 2025; Redila et al., 2021; Tatineni et al., 2010, 2019; Wong et al., 2024), suggests that one virus may interfere with the replication or movement of the other in *Ae. tauschii* or modulate host defense, leading to reduced symptom severity during co-infections. Further investigation is required to elucidate the underlying mechanisms.

Interestingly, under double-infection conditions, the correlation between symptom severity and WSMV titer was weaker and inconsistent compared to single infection (14 dpi *r* = 0.28, *p* = 0.088; 21 dpi *r* = 0.24, *p* = 0.14; 31 dpi *r* = −0.13, *p* = 0.44), possibly due to viral interference or altered host responses (Figure 8A). For TriMV, the correlation with symptom severity was weakly positive (Figure 8B), with the strongest at earlier stages (14 dpi: *r* = 0.3, *p* = 0.061; 21 dpi: *r* = 0.061, *p* = 0.72; 31 dpi: *r* = − 0.21, *p* = 0.25), suggesting that during co-infection (simultaneous infections), TriMV may drive symptom onset at early stages of the infection, while WSMV plays a more prominent role in symptom development at later stages. Tatineni et al. (2019) reported that WSMV systemic infection is facilitated in wheat pre-infected with TriMV, leading to severe disease development, while TriMV infection is negatively affected in WSMV pre-infected wheat (Tatineni et al., 2019). This contradiction reflects complex virus–virus interactions and underscores the importance of the temporal order of the infection within host tissues in determining disease outcomes.

The most significant novel locus identified in this study for WSMV severity on 5DL (~430 Mb) is particularly notable, as it contains multiple disease resistance-related genes, including *RGA4* and *RGA5* (~429 Mb), as well as several protein kinases (Supplementary Table 10). Both *RGA4* and *RGA5,* known for NB-LRR-mediated resistance to the fungal pathogen *Magnaporthe oryzae* in rice through recognition and direct binding to the pathogen avirulence (Avr) proteins (Cesari et al., 2013; Césari et al., 2014), represent strong candidates for functional validation in wheat. Interestingly, a nearby locus on 5DL has previously been associated with resistance to soilborne wheat mosaic virus (family *Virgaviridae*, genus *Furovirus*) in *Ae.* t*auschii,* with the resistant gene *Sbwm1* widely deployed in the U. S. winter wheat cultivars (Bass et al., 2006; Hao et al., 2012; Liu et al., 2014, 2020), highlighting the potential of this region for broad-spectrum viral resistance and tolerance.

Our GWAS analyses also identified loci on chromosomes 1DS and 2DS associated with symptom severity at specific stages of the infections, suggesting that multiple genes may contribute to symptom development at different stages of WSMV infections. Interestingly, the majority of loci identified here for both symptom severity and viral titers were different from the previously reported WSMV resistance loci (Chen et al., 2003; Friebe et al., 1991; Haber et al., 2006; Haley et al., 2002; Lu et al., 2011; Seifers et al., 2006), suggesting the presence of novel, uncharacterized regions.

Knowledge of genomic regions and candidate genes linked to viral titers, as well as their relationship with symptom severity, remains limited. In this study, we identified 8 loci significantly associated with WSMV replication titer, with 7 loci at 21 dpi and 1 locus at 14 dpi (Figure 9 and Table 1). The loci were distributed across chromosomes 1DS, 2DS, 5DS, 5DL, 4DS, and 7DS. Although we observed loci on 1D and 2D for both symptom severity (AUDPC) and titer, the AUDPC-associated locus was specific to the L2 lineage, whereas the titer-associated locus was detected in the L1 lineage. Notably, only one locus on 5DL was linked to both symptom severity and viral titer, suggesting that genes regulating WSMV load are largely distinct from those influencing symptom severity and disease progression. This contrasts with findings in cassava, where Nandudu et al., 2024, reported consistency between genomic regions associated with viral titers and those linked to cassava brown streak disease (CBSD) severity identified through GWAS and QTL analyses.

Known resistance genes include *Wsm1* and *Wsm3*, introgressed from *Thinopyrum intermedium* on chromosome 4DL (Danilova et al., 2017; Friebe et al., 1991), and *Wsm2*, located on chromosome 3BS approximately 14 Mb in the wheat line CO960293-2 (Haley et al., 2002; Lu et al., 2011; Seifers et al., 2006). In contrast, our study detected significant loci on chromosome 3DS at ~145 Mb (Table 1), representing a different genomic region distinct from the *Wsm2* orthologous region on 3BS. Another reported resistance source, c2652, identified in a hard red spring wheat population (Haber et al., 2006), remains largely unexplored in breeding, and no loci related to c2652 were detected in this study.

This study provides the first comprehensive phenotypic screening and GWAS analyses of *Ae. tauschii* for WSMV tolerance, revealing novel D-genome loci as valuable targets for wheat improvement. These loci offer opportunities for introgression into bread wheat via synthetic hexaploids, marker-assisted selection after validation of associated markers in wheat, and genomic prediction models to enhance selection accuracy for virus resistance, while the resistant accessions identified establish a foundation for durable resistance breeding. Future studies should expand screening to additional accessions under double-infection conditions, enabling GWAS analyses to uncover genomic regions conferring tolerance to both viruses.

## 5 Conclusion

This study reveals substantial variation for WSMV responses under single- and double-infection conditions with TriMV in *Ae.* *tauschii,* ranging from tolerant to susceptible. GWAS identified multiple loci associated with WSMV symptom severity and viral accumulation, providing potential genetic markers for marker-assisted selection. The tolerant germplasm and loci identified here offer valuable resources for pre-breeding, with further fine mapping and functional essential to enable their effective deployment in wheat improvement breeding programs.

## Data Availability

The datasets presented in this study can be found in online repositories. The names of the repository/repositories and accession number(s) can be found in the article/Supplementary material.
