# Supplementary material for: Comprehensive phenotyping and genotyping of a large diverse panel of the wheat wild relative, Aegilops tauschii for wheat streak mosaic virus tolerance
Source: Front Microbiol. 2026 Jan 16;16:1723671. doi: 10.3389/fmicb.2025.1723671 (PMC12855496; doi:10.3389/fmicb.2025.1723671)
Supplement: Supplementary file 1 [file Data_Sheet_1.docx]

***Supplementary Material***

1. **Supplementary Figures**


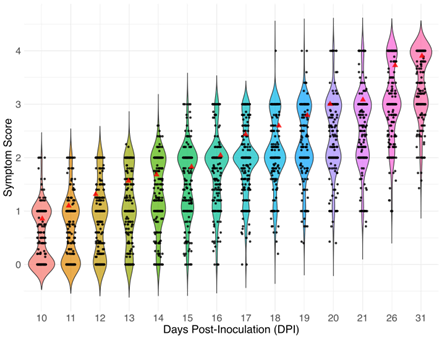


**Supplementary Figure S1:** Violin plot showing the distribution of symptom severity scores across 250 *Aegilops tauschii* accessions from 10 to 31 days post-inoculation (dpi) following single infection with Wheat streak mosaic virus (WSMV). Each dot represents the symptom score of individual accession. The red triangle indicates the symptom score of susceptible accession TA2431. Plot was generated using ggplot2 package in R (version 4.3.1). The plot illustrates a progressive increase in symptom severity over time. This trend highlights the dynamic nature of WSMV symptom development across genetically diverse accessions.


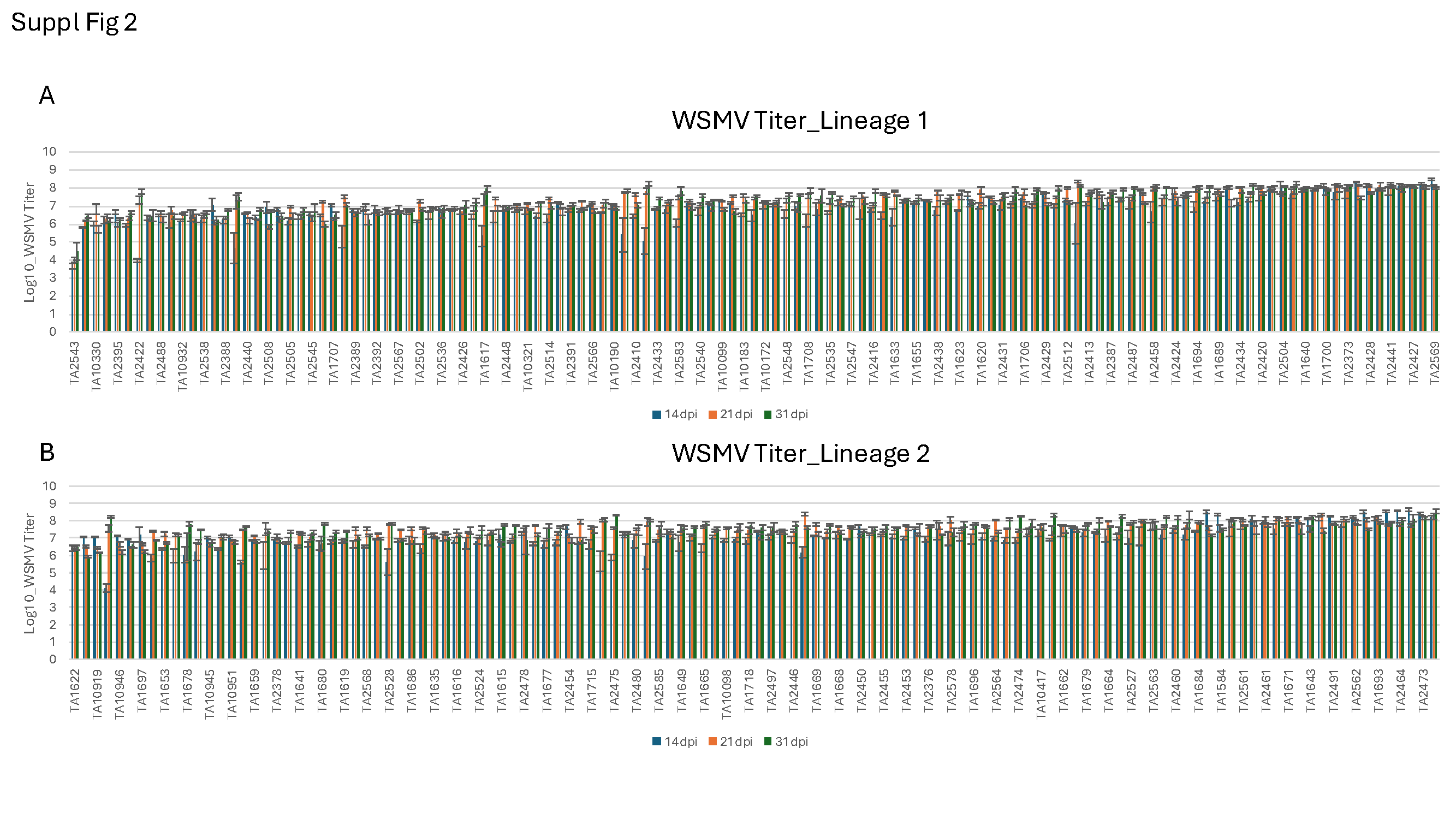
**Supplementary Figure S2:** Bar plots showing the mean Log₁₀-transformed titer of WSMV in Lineage 1 (top) and Lineage 2 (bottom) accessions of *Aegilops tauschii* under single infection. Bars represent average viral titers measured at three time points: 14 dpi (blue), 21 dpi (orange), and 31 dpi (green) with error bars indicating standard error (SE). The accessions are arranged in increasing order of mean WSMV titer.


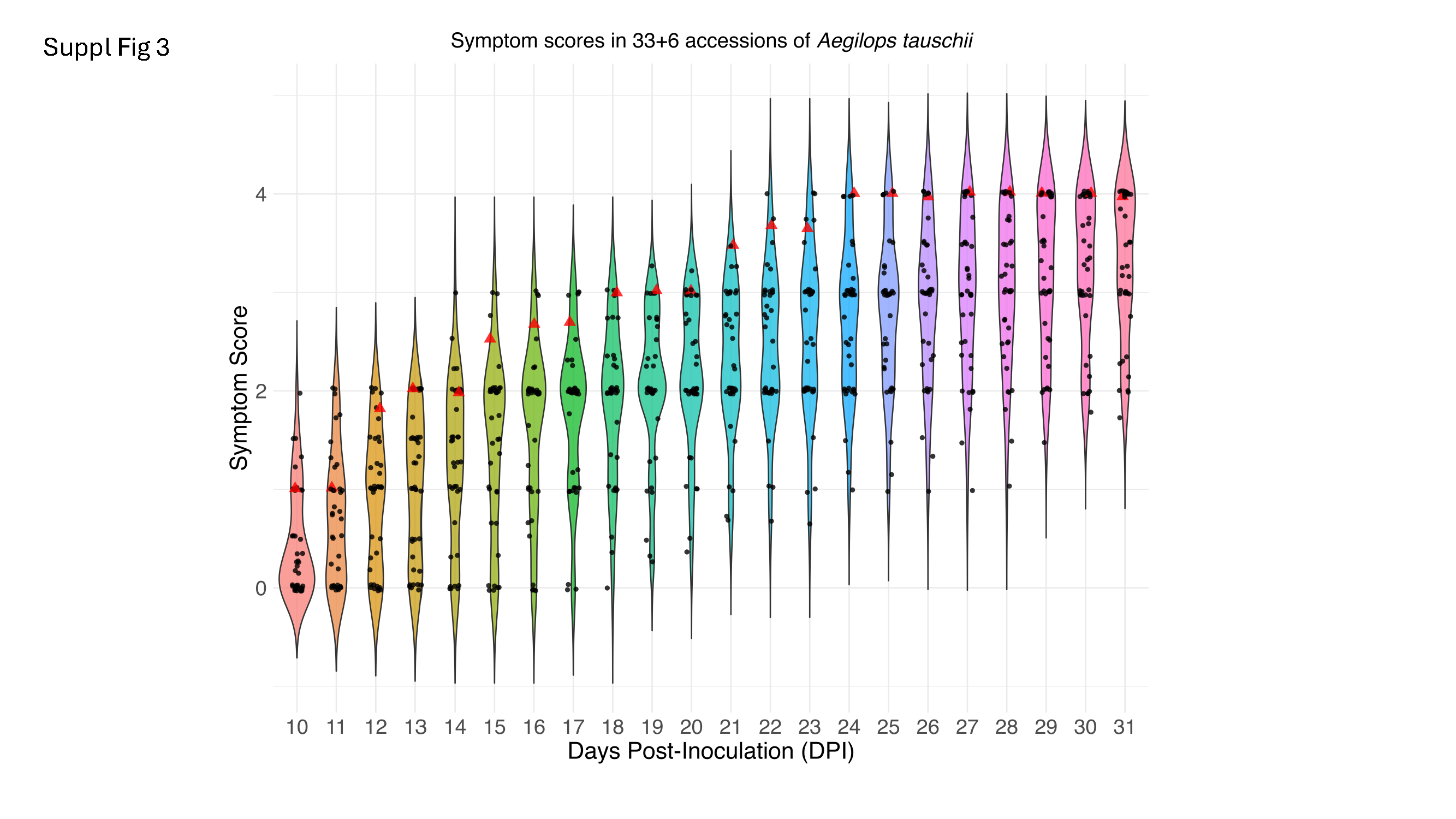


**Supplementary Figure S3:** Violin plot showing the distribution of symptom severity scores across 39 *Aegilops tauschii* accessions from 10 to 31 days post-inoculation (dpi) following mixed infection with Wheat streak mosaic virus (WSMV) and Triticum mosaic virus (TriMV). Each dot represents the symptom score of individual accession. The red triangle indicates the symptom score of susceptible accession TA2431. The plot illustrates a progressive increase in symptom severity over time. Plot was generated using ggplot2 package in R (version 4.3.1).


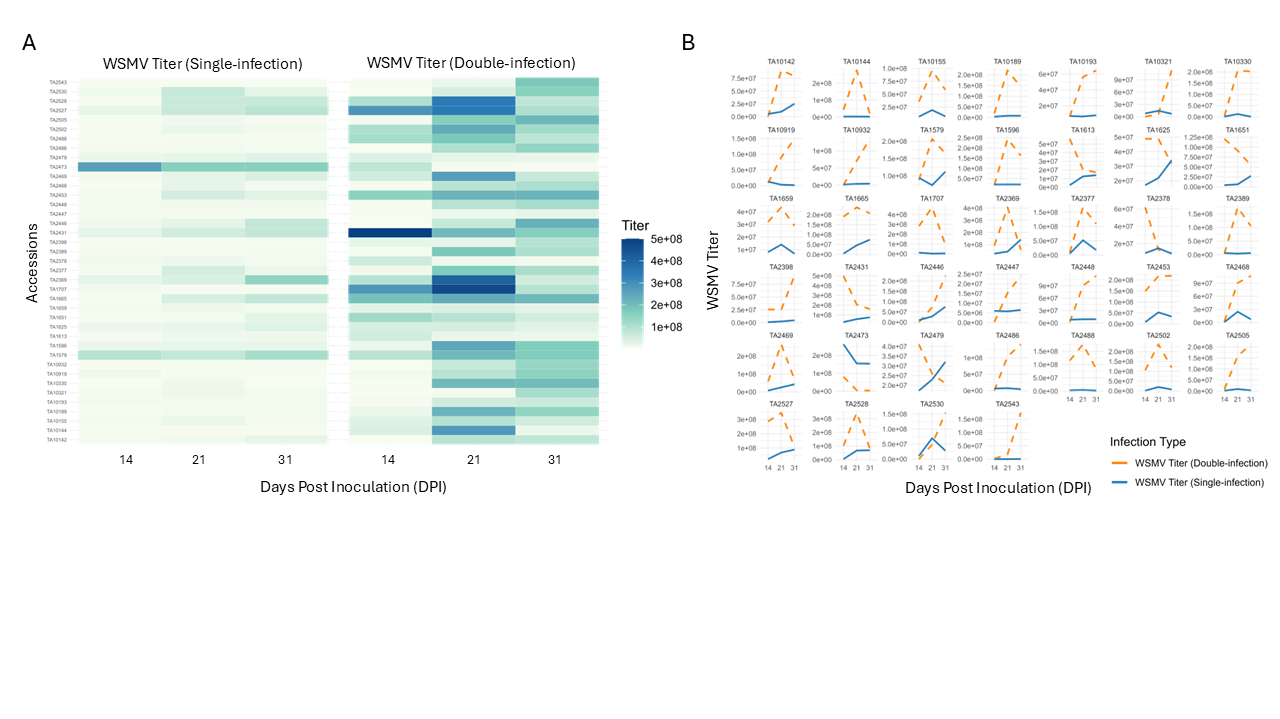


**Supplementary Figure S4. Comparison of WSMV titer in single versus mixed infections across selected *Aegilops tauschii* accessions.** (A) Heatmap showing WSMV titer values at 14-, 21-, and 31 days post-inoculation (dpi) under single (left) and mixed (right) infection conditions. Each row represents an *Ae. tauschii* genotype, and the color gradient represents the viral titer intensity. (B) Line plots comparing WSMV titer dynamics across 3 timepoints under single (red) and mixed (blue) infections for each accession tested under mixed infection. Each panel represents a unique accession. The figure illustrates differential WSMV accumulation dynamics in the presence or absence of TriMV double-infection. Plot was generated using ggplot2 and pheatmap packages in R (version 4.3.1).


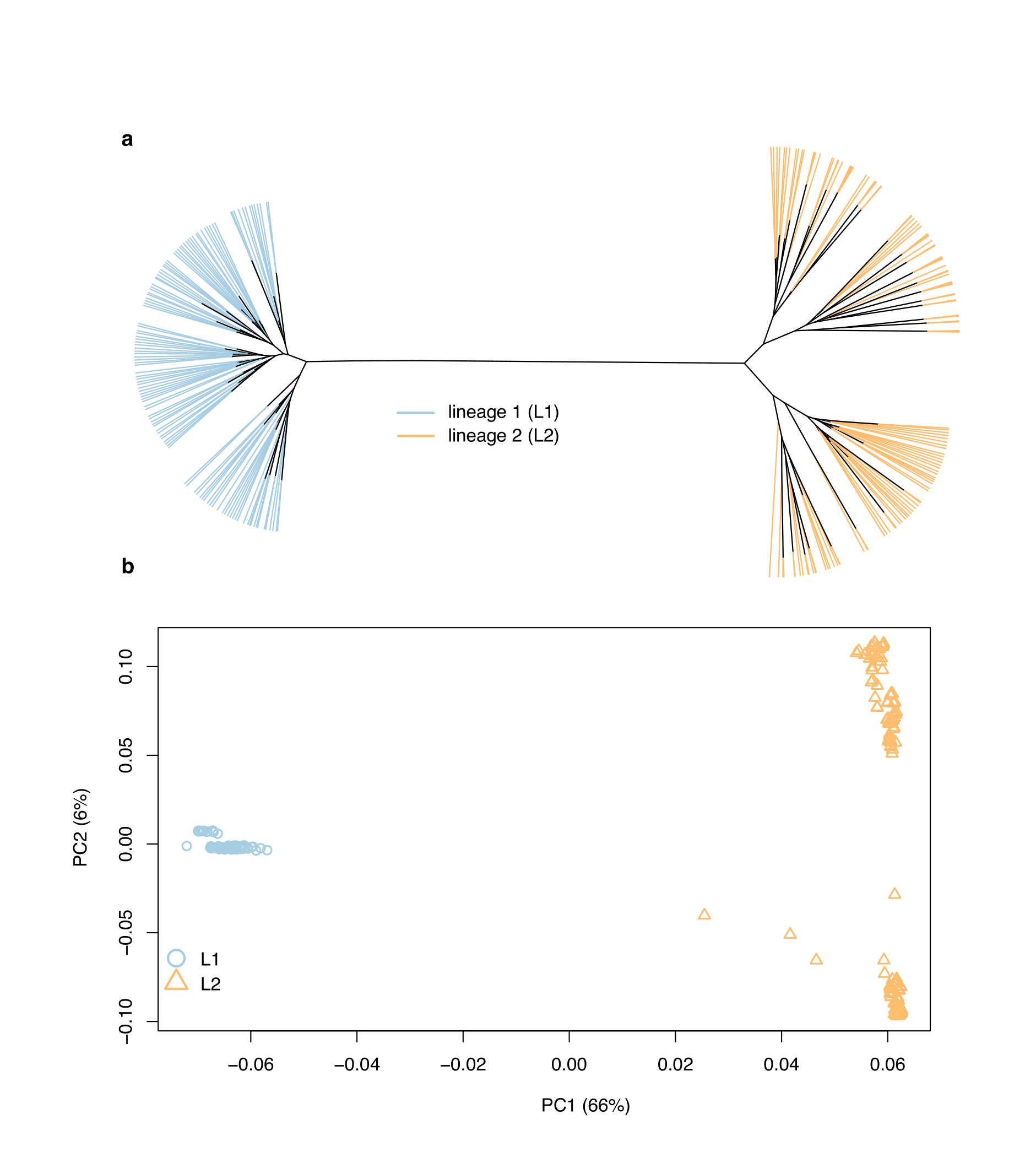

**Supplementary Figure S5. (a)** **An unrooted Neighbor-Joining tree of *Ae*. *tauschii* wild wheat in the WSMV screening panel**. The tree branches were colored based on their genetic lineages, (b) The principal component 1 (PC1) and 2 (PC2) clusters of WSMV *Ae*. *tauschii* panel. Lineage 3 in the panel had only an accession.


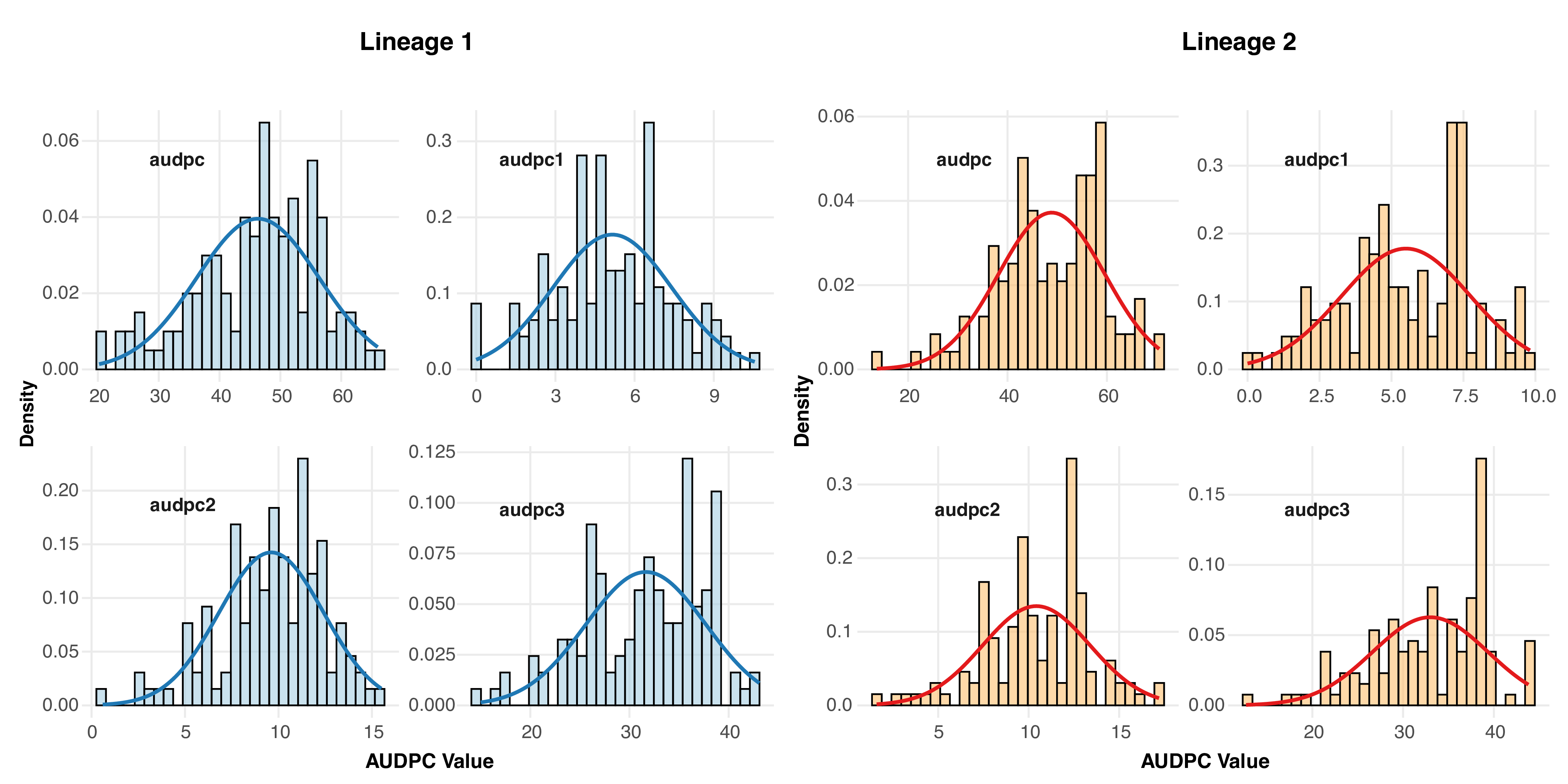


**Supplementary Figure S6. The density plot showing AUDPC values computed for the resequencing panel.** Both *Ae*. *tauschii* lineages showed near normal distributions for AUDPC values.


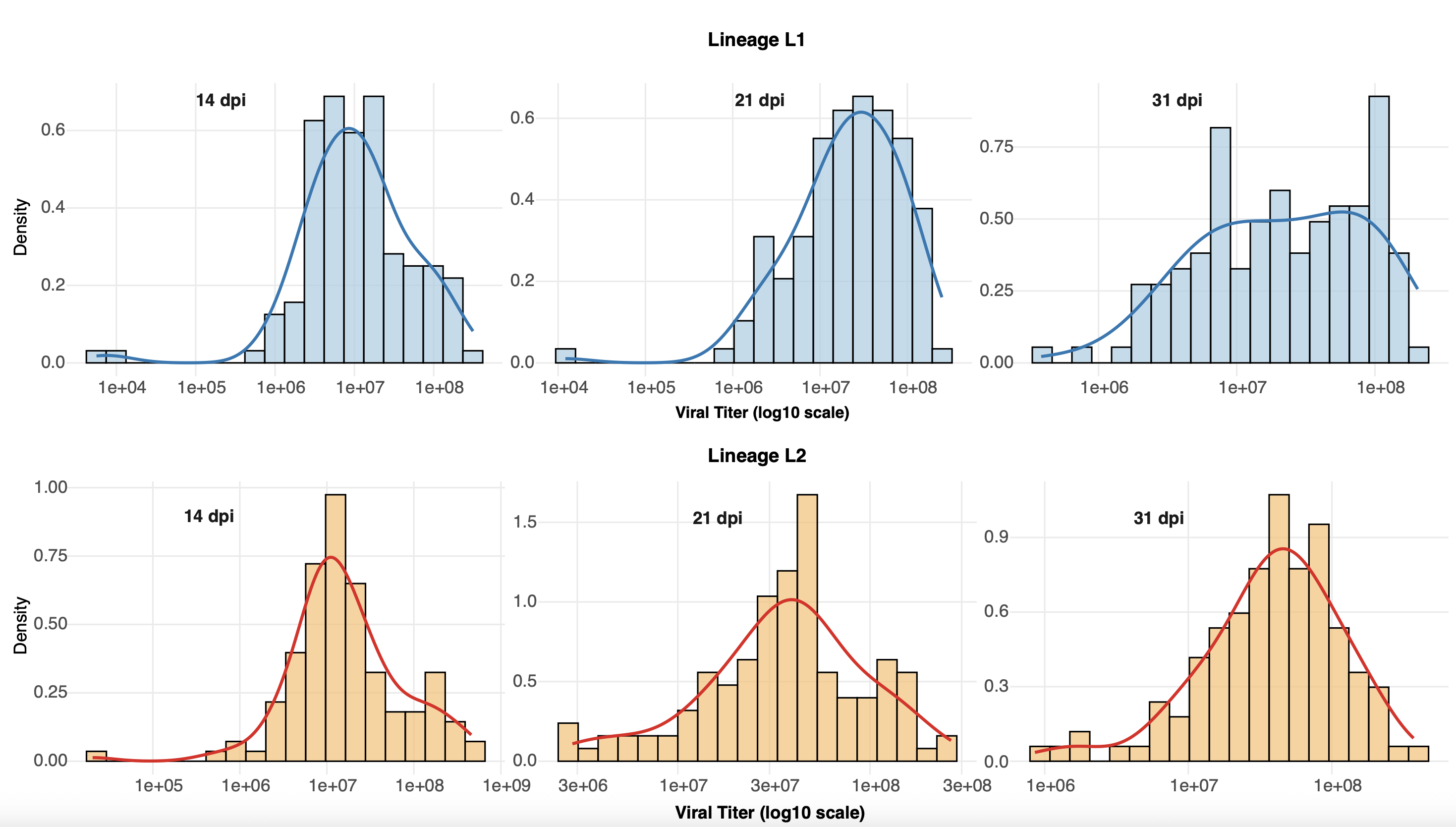


**Supplementary Figure S7. Density plot showing titer amount (log₁₀) in the resequencing panel.** Both Ae. tauschii lineages exhibited near-normal distributions of titer levels, except for Lineage 1 at 31 dpi.


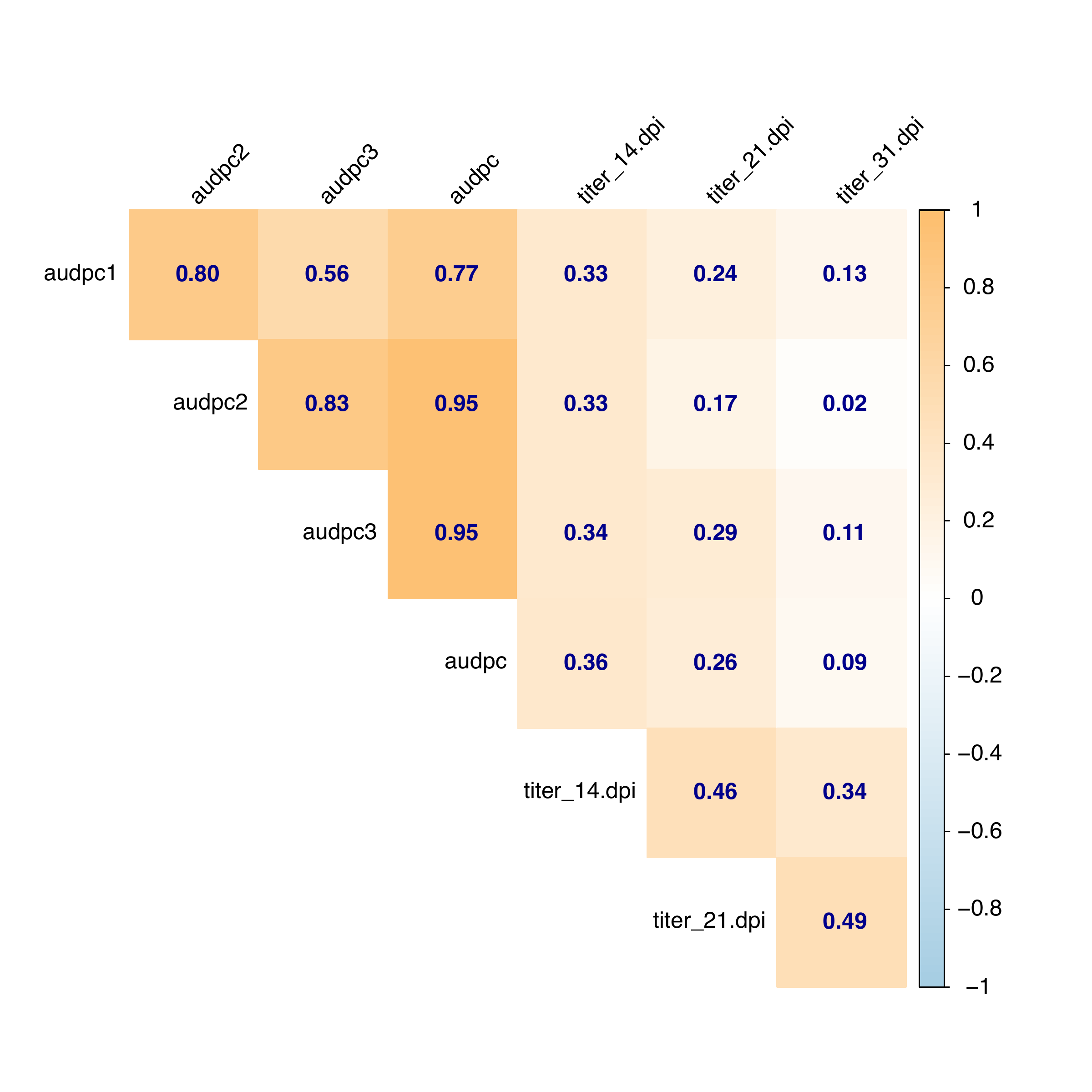


**Supplementary Figure S8. Correlation between AUDPC and WSMV titer.** The heatmap displays correlations between AUDPC values and virus titer measured at 14-, 21-, and 31 days post-inoculation (dpi).

1. **Supplementary Tables**

**Supplementary Table S1.** List of accessions screened in the study with their passport data, WSMV symptom score and titer, list of tolerant and susceptible accessions from WSMV single infection and Titer of TriMV and WSMV from mixed infection of 39 accessions (separate excel file).

**Supplementary Table S2.** Symptom score of WSMV and TriMV during Mixed infection in *Ae. tauschii* accessions (separate excel file).

**Supplementary Table S3.**Tolerance/susceptible genotypes under mixed infection (separate excel file).

**Supplementary Table S4.** Numbers of SNPs identified as raw and after filtration with provided criteria and the number of SNPs selected for the association analysis (separate excel file).

**Supplementary Table S5.** Trait values for AUDPC and viral titer used in GWAS analysis of 250 WGS *Ae. tauschii* WSMV screening panel (separate Excel file).

**Supplementary Table S6.** Gene annotation file for chromosome 5D (CBI Assembly GCF_002575655.2), obtained from NCBI (separate Excel file).
